# Supramolecular framework membrane for precise sieving of small molecules, nanoparticles and proteins

**DOI:** 10.1038/s41467-023-36684-w

**Published:** 2023-02-22

**Authors:** Guohua Zhang, Xinyue Li, Gang Chen, Yue Zhang, Mingfeng Wei, Xiaofei Chen, Bao Li, Yuqing Wu, Lixin Wu

**Affiliations:** State Key Laboratory of Supramolecular Structure and Materials, College of Chemistry, Jilin University, Changchun, 130012 P. R. China

**Keywords:** Supramolecular polymers, Self-assembly, Molecular self-assembly

## Abstract

Synthetic framework materials have been cherished as appealing candidates for separation membranes in daily life and industry, while the challenges still remain in precise control of aperture distribution and separation threshold, mild processing methods, and extensive application aspects. Here, we show a two-dimensional (2D) processible supramolecular framework (SF) by integrating directional organic host-guest motifs and inorganic functional polyanionic clusters. The thickness and flexibility of the obtained 2D SFs are tuned by the solvent modulation to the interlayer interactions, and the optimized SFs with limited layers but micron-sized areas are used to fabricate the sustainable membranes. The uniform nanopores allow the membrane composed of layered SF to exhibit strict size retention for substrates with the rejection value of 3.8 nm, and the separation accuracy within 5 kDa for proteins. Furthermore, the membrane performs high charge selectivity for charged organics, nanoparticles, and proteins, due to the insertion of polyanionic clusters in the framework skeletons. This work displays the extensional separation potentials of self-assembled framework membranes comprising of small-molecules and provides a platform for the preparation of multifunctional framework materials due to the conveniently ionic exchange of the counterions of the polyanionic clusters.

## Abstract (web summary)

Synthetic framework materials are appealing candidates for the fabrication of separation membranes but realizing precise control of aperture distribution and separation threshold remains challenging. Here, the authors show a two-dimensional processible supramolecular framework which can be used in the fabrication of separation membranes by integrating directional organic host-guest motifs and inorganic functional polyanionic clusters.

## Introduction

Membrane separation has become one of the most promising approaches for purification, extraction, permeation, concentration, and so forth in the fields of chemistry, biology, energy, and environmental sciences, because of the simplicity of devices, easy operation, and wide applicability in gas, liquid, and interface systems^1–5^. Various organic and inorganic molecular assemblies that give stability, selectivity, and permeance, have been devoted to strengthening the structure and separation properties of the membranes^6^. Among them, synthetic framework materials like metal–organic frameworks (MOFs) and covalent organic frameworks (COFs) as well as porous aromatic frameworks, are mostly adopted due to their uniform and tuneable nanopores with high selectivity^7–10^. However, the synthetic framework materials usually take the shape of insoluble and intractable crystalline powders at the micrometer scale^11–15^ and require the support of polymer matrixes to assist in film formation for avoiding possible defects during processing^16–19^. Although the added polymers improve the structural property and fill the chinks between porous particles greatly, the unfavorably interfacial slits between the dispersed framework materials and polymer-matrix and the relatively limited loading capacity due to particle agglomeration, impede the performance of membranes to achieve Robeson’s upper bound^20^. Alternatively, highly laminated graphene oxide membranes exhibit outstanding sieving properties accompanied by ultrafast organic solvent permeation^21^, and other two-dimensional (2D) ultrathin nanosheet-like materials (graphdiyne, MXene, transition metal dichalcogenides, etc.) have been considered as attractive building blocks to construct high-performance separation membranes because of their monoatomic thickness, high mechanical strength, and ability to stack nanochannels in their lattices^22^. Such flexible single molecular layer dispersion and the 2D packing structure make the polymer matrix support no longer inevitable because the slits existing between layers and at the boundary become the nanochannels for size-screened separations. Actually, the separation process in the method depends closely on the defect control of nanosheet buildings and/or the layered packing channels, so it is still hard to control the pore size and achieve high porosity in large-area, and the sophisticated modification of nanochannels making them mostly use for size-exclusive separation. Thus, it is still highly desired to develop an alternative route to construct porous nanosheet materials with uniform apertures, easy modification, and convenient processing, for the use in separation membranes with fewer defects to meet on-demand applications.

Processible 2D framework materials have become satisfying candidates as selective layers for preparing the separation membranes with improved permeability and selectivity^23–26^. Beyond the coordination and covalent bindings, non-covalent interactions such as host-guest inclusion, hydrogen bond, and electrostatic interaction have been demonstrated as the applicable driving forces in the construction of ordered porous structures, like hydrogen-bonded organic frameworks (HOFs)^27^, supramolecular organic frameworks (SOFs)^28^, and ionic organic-inorganic frameworks (IOIFs)^29^. While bearing rigid porous networks, the supramolecular frameworks (SFs) appear more attractive advantages in terms of structural flexibility, functional programmability, and environmental adaptability, which provide a potential platform for exploring advanced function separation materials^30–33^. However, the size of most supramolecular assemblies is nano-scaled and positively associated with concentration^34–36^, meaning that dilution will disassemble or destroy them, which precludes their direct uses in membrane separation. Thus, if the large-area nanosheet assemblies become available with improved structural strength, the reduced cracks and defects of framework pores, they will be prone to process the SFs with inherent nanopores into robust membranes for diverse function separations.

**Fig. 1:**
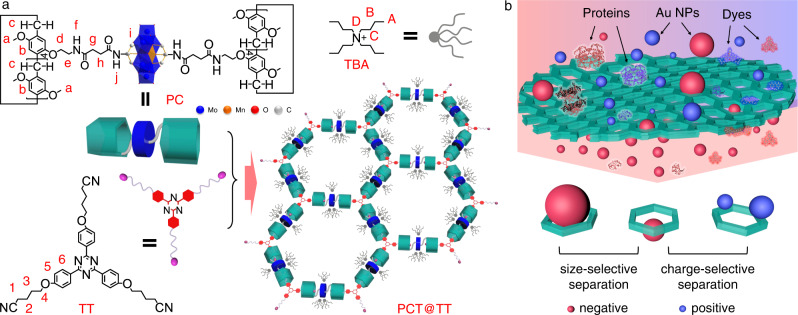
Fabrication of SF assembly and its membrane separation performance. **a** Schematic illustration for the formation of the SF structure via host-guest interaction. **b** Size and charge dual-selective separation of Au NPs, dyes, and proteins using SF membrane. The red uppercase and lowercase letters in PC, TBA, and red numbers in TT are used to label the positions of hydrogen atoms, which are cited in the following text and Fig. 2.

The combination of polyoxometalate (POM) and organic macrocyclic molecules gains the organic-inorganic hybrid macrocyclic hosts with precise molecule composition and structure, which shows important modules for the preparation of the accurate and expanded assemblies driven by microphase separation and multiple interactions^37–41^. In recent studies, we have demonstrated that the SFs, constructed by inorganic polyanionic clusters and organic host-guest modules through electrostatic and host-guest interactions, can be processed into membranes for on-demand liquid-liquid separation^30^ and precise size-dependent separation^31^. Compared with the direct synthesis of a framework structure, the route of the assembly via the formation of pseudorotaxane not only reduces the difficulty of synthesis but also brings structural flexibility and disassembly of the framework that facilitates the self-healing and recovery of the membranes. But basically, the multi-functionalized separations for both molecules and nanoparticles based on the principles of surface adsorption and size exclusion are not yet achieved in one SF membrane up to date. In this context, we herein report a processible SF assembly constructed by polyanionic POM cluster grafting pillar[5]arene host groups and neutral tritopic guest molecules via multiple host-guest interactions (Fig. 1a). The micro-scale and flexible 2D SF nanosheets are dispersed in solutions in the form of thin or single layers homogeneously via solution exfoliation and further processed into separation membranes under suction. The negatively charged and hydrophilic POMs in the framework not only allow the membrane to be permeated by water but also impart it with charge-selective capacity. Importantly, the open hexagonal pores within the single-layer honeycomb-like framework structure provide uniform channels for the size and charge dual-selective separation of Au nanoparticles (NPs), dyes, and proteins with a strict cutting-off value via a simple filtration (Fig. 1b). These results unfold a seductive opportunity for flexible framework membranes in highly efficient and energy-saving purification and separation of biomolecules and synthetic polymers.

## Results

### Module synthesis

The doubly pillararene-grafted cluster (PC) host in a linear shape is synthesized through the reaction of mono-aminoethoxy methoxypillar[5]arene, prepared by the amination of its mono-ethoxyl methoxypillar[5]arene bromide, with an N-hydroxy succinimide ester activated POM cluster ([Mn(OH)_6_Mo_6_O_18_]^3−^) following a similar procedure in literature^42^. The formed final product in tetrabutylammonium (TBA) salt (abbrev. as PCT) is characterized by proton nuclear magnetic resonance (^1^H NMR), mass spectra (MS), Fourier transform infrared (FT-IR) spectra, and elemental analysis. The product is soluble in chloroform, and dimethyl sulfoxide (DMSO) easily, but insoluble in ethanol, ethyl acetate, and water. The TBA counterions can be replaced with other functional cations easily through mono- or double-phase ion-exchange reactions^43^. Tritopic guest molecule (TT) and monotopic guest molecule (MT) are synthesized by the reaction of triphenol-triazine or 4-methoxyphenol with 5-bromophetanenitrile, respectively. Methyl pillar[5]arene (MP) is obtained via published procedures^44^. The synthetic routes and detailed characterizations of all modules are summarized in the Electronic Supplementary Information (Supplementary Figs. 1–16, Supplementary Table 1).

### Host–guest interaction of PCT host and TT guest

**Fig. 2:**
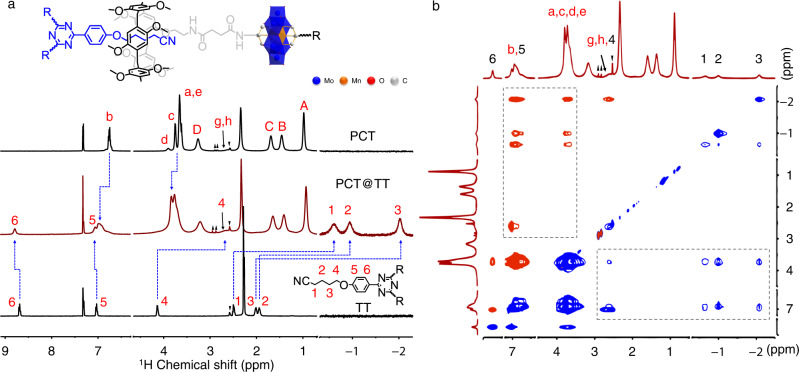
Host–guest interaction between PCT and TT. **a** Partial ^1^H NMR spectra (CDCl_3_/DMSO-*d*_6_ = 50:1, 500 MHz, 25 °C) of TT, PCT@TT (3:2), and PCT. **b** 2D NOESY NMR spectra (CDCl_3_/DMSO-*d*_6_ = 50:1, 500 MHz, 25 °C) of PCT@TT (3:2). Reverse triangle is assigned to solvent DMSO, and the triangle points to the peaks of residual N,N-Dimethylformamide (DMF).

The binding behavior of PCT and TT is systematically evaluated. Both isolate host complex and guest molecule dissolve in CHCl_3_ easily. However, the insoluble assemblies start to appear and float on the surface of the solution after mixing the two components together for a while, showing direct evidence of the interaction between the two compounds. Afterward, ^1^H NMR experiments are performed in CDCl_3_/DMSO-*d*_6_ (v/v = 50:1) mixed solvent, which gives a clear observation of the chemical shifts due to the enhanced solubility after adding DMSO. The proton peaks of the mixture appear obviously shift and broaden in comparison to the isolated components (Fig. 2a) due to the inclusion-induced shielding effect^45^, confirming the host-guest complexation. The proton peaks H(1–4) ascribing to guest TT shift up-field greatly, indicating the alkyl chain moiety is included inside the cavity of pillar[5]arene group. Meanwhile, the peaks H(5–6) move down-field, showing that the phenyl part is located outside of the pillar[5]arene group. The 2D nuclear overhauser effect spectroscopy (NOESY) NMR study of PCT host and TT guest further identifies the spatial positions of the protons in the host-guest inclusion complex (Fig. 2b). The correlations between H(1–4) of TT and H(a–d) of PCT are observed clearly, further demonstrating that the alkyl chains of TT threads into the cavity of pillar[5]arene to form the pseudorotaxanes^46^. The ^1^H NMR titration experiment (Supplementary Fig. 17) displays a slow chemical exchange on the NMR time scale^47^, by fixing TT at a concentration of 4.0 mM and varying the concentration PCT. Upon the addition of PCT, the integral values of proton H(1–4) on uncomplexed TT decrease obviously, while the signals belonging to included TT appear and get enhanced due to the formed inclusion complex. Such an inclusion change comes to a constant state after the addition of 1.5 equivalent of PCT, confirming that the binding ratio of PCT to TT is 3:2. The FT-IR spectrum of the included product in precipitate shows the characteristic vibrations of both PCT and TT (Supplementary Fig. 18). The organic elemental analysis result also points out a 3:2 combination of PCT and TT (Supplementary Table 2). According to the isothermal titration of calorimetry (ITC) result (Supplementary Fig. 19), the binding constant for the pillar[5]arene and cyan alkyl chain is calculated to be ca. 740 ± 26.9 M^−1^ in CDCl_3_, in the case of model compounds MP and MT. Thus, a stable “3 + 2” type inclusion reaction can be inferred.

### Co-assembly and framework structure

**Fig. 3:**
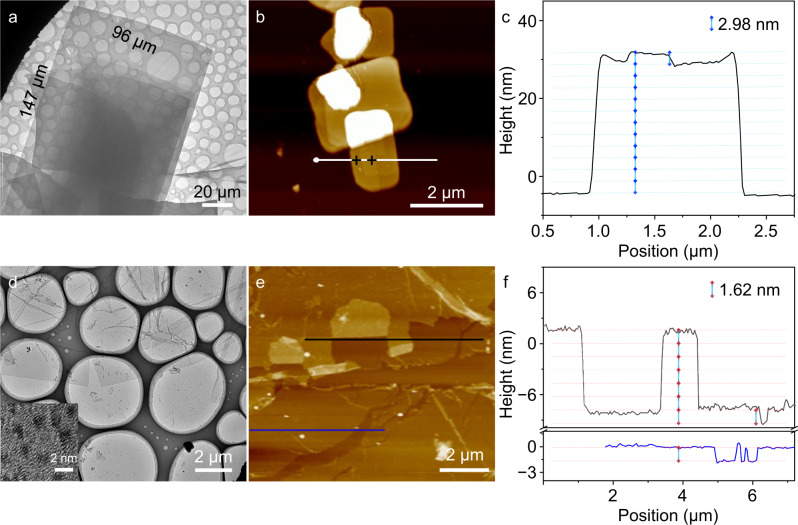
Co-assembly of PCT and TT and exfoliated SF assemblies. **a** TEM and **b** AFM images of PCT@TT assemblies in CHCl_3_ and **c** height profile analysis measured across the white line in Fig. 3b. **d** TEM with an inset of local amplification and **e** AFM images of exfoliated SF assemblies in DMSO/H_2_O (1:4 v/v) and **f** corresponding height profile analysis of across the black and blue lines in Fig. 3e.

Compared with the sphere-like assembly of isolated PCT in CHCl_3_ (Supplementary Fig. 20), the transmission electron microscopic (TEM) and scanning electron microscopic (SEM) images of inclusion product PCT@TT show the rectangular sheet-like morphology at micrometer scale (Supplementary Fig. 21a, b). This result indicates that the supramolecular polymerization upon host-guest inclusion extends within the 2D plane. The length of one side in the nanosheet even reaches more than 140 μm (Fig. 3a) and is easily discerned under an optical microscope (Supplementary Fig. 21c) after 10 h of incubation, revealing a very high degree of supramolecular polymerization. The high-resolution TEM image of the nanosheet assembly figures out the existence of particles with a size of about 1.0 nm (Supplementary Fig. 22a), which can be ascertained as Anderson POMs due to their high electron density. The irregularly dispersed POM should be sourced from the thermal drift during electron beam focusing. The selected energy dispersive X-ray (EDX) spectrum (Supplementary Fig. 22b) on the nanosheet assemblies gives the distributions of Mn and Mo elements, suggesting the existence of the inorganic clusters.

Noticeably, the regular nanosheet morphology of the formed A_2_B_3_ type supramolecular polymers in this work is much different from the reported ones which preferentially show classic Cayley tree-like hyperbranched structures^48–50^. Instead, a highly ordered cross-linked structure can be speculated consciously. The reason is that the rigid structure of POM can act as a barrier for molecular entanglement, which is helpful to form the framework structure other than the hyperbranched growth. The single crystal X-ray diffraction (SCXRD) in powder mode (Supplementary Fig. 23a) presents multiple diffraction peaks, and the peaks (2θ) at 3.20°, 4.30°, 5.15°, 6.70°, 7.05°, and 7.90° can be well fitted to the 200, 210, 300, 400, 320, and 410 diffraction indexes of a hexagonal packing model with a value of *a*-axis of 6.37 nm corresponding to a side length of 3.68 nm, which is calculated from the interplanar spacing *d*_200_ value (Supplementary Table 3). Grazing incidence X-ray diffraction (GIXRD, incidence angel: 2°) of PCT@TT SF membrane gives clear separated diffraction peaks with the appearance of 100 facets (Supplementary Fig. 23b), indicating the SF membrane is crystalline well with a 2D extending layer parallel to the substrate^51^. Combining the binding ratio and topological structure of building units PCT and TT, and the indexation of XRD data, a 2D hexagonal framework structure of the A_2_B_3_ supramolecular polymer can be inferred. By collecting the size of each component, when the flexible linking group adopts a restricted state, the estimated size of the hexagonal framework is 3.77 nm, which closely matches the calculated value of SCXRD (Supplementary Fig. 24). Thus, the framework should have an approximate pore size of 4.03 nm in diameter. To further figure out the layered structure, the SF assemblies are spread on a clean silicon wafer and the atomic force microscopic (AFM) image shows the morphology of assemblies with height steps of the smallest distance ca. 2.98 nm (Fig. 3b, c). Those lamellar assemblies with larger heights matching to integer folds of the smallest one indicate the formation of multilayers. However, considering that the pillar[5]arene with an external diameter of 1.12 nm adopting a slip double-layer packing model in pillar[5]arene derivative crystals has a packing distance of 0.37 nm^44^, the observed experiment distance is very close to the vertical dimension of the double-layered structure (2d = 3.02 nm) (Supplementary Fig. 24). Apparently, in comparison to a single layer, multi-layered packing is more favorable for decreasing the interfacial energy because the exposure of the hydrophilic POM clusters in a hydrophobic media can be diminished to the minimum at the situation of tight overlapping between adjacent layers. The hydrophilic properties of POMs reduce the solubility of the SFs in hydrophobic media but promote their dispersion in hydrophilic solvent simultaneously.

### Solvent exfoliation and stability of SFs

Although 2D porous materials possess indispensable merits in building separation systems, the control of the third dimension is still necessary to obtain the processable properties and restrain the adverse effect from inner layer defects as well as interlayer slits^52^. Besides direct preparation, liquid exfoliation^53^, and mechanical delamination^54^ are common procedures to make the 2D porous materials thinner for the preparation of compact membranes. Thus, we get the 2D flexible SFs with fewer layers or even a single layer by controlling the liquid exfoliation strategy. In this case, the liquid environment needs to ensure that only the layered interaction is weakened while the in-plane interaction maintains. Here, the in-plane interaction is mainly host-guest interaction, and it is stable in most non-polar and weakly polar solvents, while the layered stacking can be taken as the hydrophilic POM clusters tending to stack densely in a hydrophobic environment. As a result, we exfoliate the SFs in the mixture of CHCl_3_ with other polar solvents firstly, since CHCl_3_ can preserve the host-guest interactions in-plane and the polar solvents may decrease the interlayer interactions. Although maintained in the mixed solvents such as CHCl_3_/isopropanol (IPA), CHCl_3_/ethyl acetate (EA), CHCl_3_/tetrahydrofuran (THF), and CHCl_3_/acetone with a volume ratio of 25:1, the prepared nanosheets in the TEM images still have high contrast (Supplementary Fig. 25), indicating that these mixed solvents cannot weaken the interlayer interactions well. In contrast, the mixed solvents like CHCl_3_/methanol, CHCl_3_/DMF, and CHCl_3_/DMSO at the same volume ratio make the solutions transparent, implying the destruction or disaggregation of SF assemblies. These results point out the stability difference of the prepared nanosheets in various environments and the appropriate application range of series solutions.

The hydrophilic POMs homogeneously dispersed on the nanosheets reduce the solubility of the SFs in a hydrophobic media but promote their dispersion in a hydrophilic solvent simultaneously. As the host-guest interactions of PCT and TT still exist in DMSO/H_2_O mixed solvent (Supplementary Fig. 26), and the two hydrophilic solvents are beneficial to the dissociation of the POM clusters located in neighboring layers driven by the interfacial energy, we evaluate the morphologic change of SFs in the mixed solvents with different volume ratios. Fortunately, when the volume ratio of H_2_O to DMSO reaches 4:1, the TEM images show that the nanosheets keep at complete morphology but bear a relatively low contrast, indicating the successful exfoliation of SF nanosheets (Fig. 3d). The magnified image displays the POM clusters distributed on the nanosheet (Supplementary Fig. 27a), although they are not in good hexagonal order due to the thermal drift caused by electron beam focusing. The PXRD data (Supplementary Fig. 27b) suggest a similar but relatively less ordered structure of the SFs because of the inaccurate packing of thin layers obtained by exfoliation^55^. The AFM measurement (Fig. 3e, f) demonstrates the formation of a layer with a thickness of ca. 1.62 nm, close to the calculated ideal length of a single layer. Moreover, the thinner SF nanosheet assemblies exfoliated by the DMSO/H_2_O mixed solvent become more flexible than those prepared from CHCl_3_, which facilitates further processing into membranes.

### Preparation and characterization of separation membranes

On account of the flexible feature of exfoliated SF assemblies obtained in DMSO/H_2_O mixtures, the membranes are prepared for further separation functions. As depicted in Experimental Section, the membrane is prepared through filtration under gradient-reduced pressure using commercial porous polymer membranes as supporting substrates. Such prepared membranes perform flexibility and no obvious cracks are observed from the digital photo and SEM image (Supplementary Fig. 28). The thickness of the membrane from several micrometers to several tens of micrometers can be tuned by modulating the volume of the SF assembly solution at a fixed concentration (Supplementary Fig. 29). The layered packing of SF assemblies is confirmed from the lateral view of the SEM images (Supplementary Fig. 30). After the filtration, no absorptions ascribing to the residue SF assemblies or the dissociated building units in the filtrate are checked out from the UV-vis absorption spectra (Supplementary Fig. 31), indicating the robustness of the membranes under the reduced pressure. To demonstrate the porosity of the prepared membrane, the N_2_ adsorption isotherm experiment is carried out and analyzed by means of the density functional theory method, which gives the most probable pore size distribution of 3.78 nm (Supplementary Fig. 32). Although the measured volume of pores seems small like the case in published results^56^, the similar value to the estimated pore size (4.03 nm) of the proposed hexagonal framework (Supplementary Fig. 24) confirms that the prepared membrane retains the nanopores of SFs effectively.

### Size-dependent separation for Au NPs

**Fig. 4:**
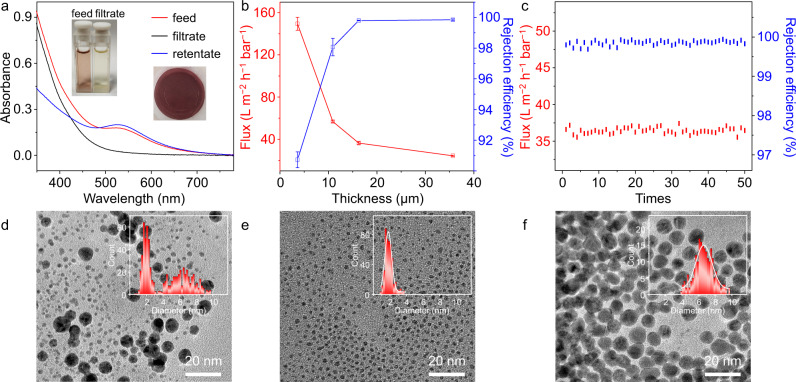
Separation of Au NPs by SF membrane. **a** UV-vis spectra of Au NPs in feed, filtrate, and retentate solutions with insets of photographs of solutions and the used membrane. **b** Flux and rejection efficiency for Au NPs versus the increase of membrane thickness. Data are collected to get average values (*n* = 5). **c** Flux and rejection efficiency for Au NPs versus reused filtration cycles. **d**–**f** TEM images of Au NPs with corresponding size histograms as insets, **d** feed, **e** filtrate, and **f** retentate.

The artificially synthesized inorganic NPs exhibiting narrow polydispersity make them suitable models for evaluating the pore size of the separation membrane because their shapes do not change during the separation process. Importantly, the narrow dispersion of inorganic semiconductor NPs is more favorable for their excellent performance in practical applications. To examine the performance of the SF membranes for size-selective separation, we prepare the mercaptopropionic acid (MPA) stabilized Au NPs with different size ranges and mix them to get a feed solution with a size distribution of about 1.5–10 nm (Supplementary Figs. 33–34). The distinct color change between the feed and the filtrate gives direct evidence that the membrane intercepted larger NPs during the separation process (Fig. 4a, Supplementary Movie 1). Compared to the feed solution with the absorption band of 524 nm, which belongs to the surface plasmon resonance (SPR) band of larger NPs, the UV-vis spectrum of the filtrate shows the absence of the absorption, confirming the location of smaller NPs. However, the retentate solution displays a distinct SPR peak at the same position, suggesting the successful interception of larger NPs. As shown in Fig. 4b, the rejection efficiency of larger size NPs for membrane thickness of ca. 3.6 μm is calculated to be ca. 90.7% with a permeation of 149.5 ± 6.2 L m^−2^ h^−1^ bar^−1^ under reduced pressure (−0.08 MPa). By increasing the membrane thickness over 16.5 μm, an enhanced rejection efficiency of over 99.8% is obtained with a flux decreasing to ca. 36.4 ± 1.2 L m^−2^ h^−1^ bar^−1^. After a sonication of the used membrane in water, the rejected Au NPs redisperse in the solution while the SF assemblies precipitate at the bottom. The precipitated SFs are collected by centrifugation and reused to reprepare the membrane to check the recyclability of the SF assemblies. As shown in Fig. 4c, even after 50 cycles of repeated filtrations, the permeability and rejection efficiency of the remade membrane still shows a quite stable separation performance, indicating the well-sustainable utilization of the SF assemblies. The TEM images (Fig. 4d–f) show that only the smaller Au NPs are left in the filtrate and most of the larger particles are rejected. The statistical result of corresponding particle sizes indicates a distinct rejection window of 3.8 nm. Notably, the interception value is a little smaller than the aperture estimated from XRD and N_2_ adsorption isotherm analysis, possibly due to the neglect of the molecular length of the stabilizer ligand under TEM statistics. These results indicate the consecutive sieving ability of SF membranes for nanoparticles without any obvious leakages, and it can be inferred that the continuous rejection of NPs with critical size value is conducted by the framework structure. As a result, the window value can be regarded as a reference for the following size-dependent separations.

### Charge-dependent separation for dyes and Au NPs

**Fig. 5:**
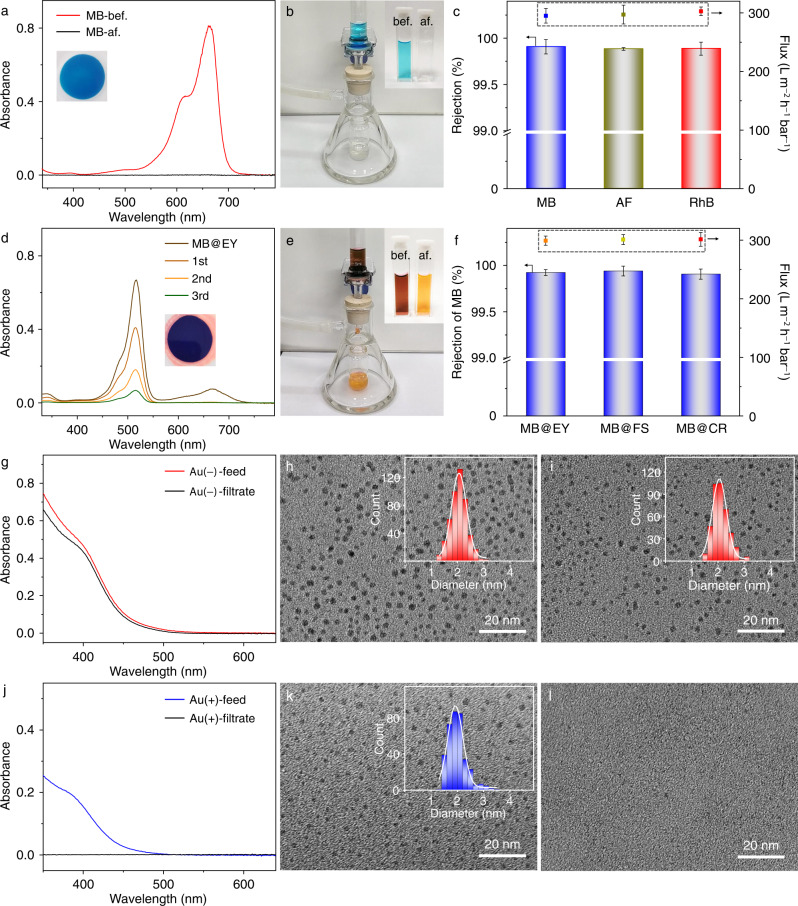
Separation of charged dyes and Au NPs. **a** UV-vis spectra and **b** digital photo of MB before and after separation. **c** Filtration performance for different cationic dyes. **d** UV-vis spectra and **e** digital photo of MB@EY mixture before and after separation. **f** Filtration performance for cationic MB and different anionic dye mixtures. Data are collected to get average values (*n* = 5). **g** UV-vis spectra and TEM images of Au(−) NPs **h** before and **i** after separation. **j** UV-vis spectra and TEM images of Au(+) NPs **k** before and **l** after separation. The insets in **h**, **i**, and **k** are their corresponding size histograms.

Generally, the separation of rigid particles based on size screening is much easier to achieve than charged-based separation, which performs better in systems when electric nanopore-particle interactions become significant^57^. The POM complexes often show a negative surface potential that can absorb cationic molecules for further applications due to the unsaturation of delocalized electrostatic interaction^58, 59^. To confirm the surface charge of the SFs, Zeta potential measurement of the SF assembly in DMSO/H_2_O solution is conducted and it gives a negative surface potential of −32.6 ± 1.7 mV (Supplementary Fig. 35), as observed in other organic cation-enwrapped POM complexes^60^. In such cases, cationic dyes are chosen to examine the nanofiltration performance of SF membranes for the removal of ionic organic molecules, which is important in the water treatment and dyestuff industry. The separation experiments are performed by using a membrane with a thickness of about 16.5 μm for the filtration under reduced pressure (−0.08 MPa). By taking methylene blue (MB) as a model molecule (Fig. 5a, b, Supplementary Movie 2), in comparison to the blue color of the feed solution, the filtrate becomes nearly colorless, indicating the successful interception of cationic dye MB. The rejection efficiency, calculated from the characteristic absorbance of the band at 668 nm before and after filtration, is over 99.9%. ^1^H NMR spectrum of the filtrate does not show the signals of TBA cations (Supplementary Fig. 36), implying that the primary counterions surrounding the cluster are not replaced by the captured MB cations. Because the molecular size of MB is much smaller than the channels of the framework assembly, the separation capacity of the membrane should be a result of the electrostatically unsaturated adsorption of polyanionic clusters. In an experiment for 10 days of continuous and interrupted separation ([MB] = 5 × 10^−6^ mg/L), the rejection efficiency maintains unchanged essentially but ca. 0.9% decrease of the flux on the 10^th^ day (Supplementary Fig. 37), demonstrating the persistent mechanical strength and pore robustness of the membranes along with the filtration process under the reduced pressure. To identify the regularity for the separation of other cationic dyes, acriflavine (AF) and rhodamine B (RhB), are then used in the filtration under the same conditions (Supplementary Fig. 38). Consistent with the interception behavior of MB, both AF and RhB are blocked by the SF membrane with rejection efficiencies over 99.9% (Fig. 5c), proving the universality of the membrane to remove cationic dyes. The permeability for dyes is significantly enhanced to 299.1 ± 7.9 L m^−2^ h^−1^ bar^−1^ than the Au NPs, which may be due to the fact that the diffusion coefficient is inversely proportional to the molecular/particle size^61^.

In contrast to cationic dyes, the SF membrane shows an opposite nanofiltration performance for anionic dyes such as eosin Y (EY), fluorescein sodium (FS), Congo red (CR), etc., which pass through freely (Supplementary Movie 3). This result is also in accordance with the process of charge-dependent separation. To identify the effectiveness of the membrane separation, the mixture of cationic dye MB and anionic dye EY (Fig. 5d–f, Supplementary Movie 4) at the condition without charge neutralization-induced flocculation is applied. The dramatically contrastive color change demonstrates the accessible separation of dyes with opposite charges. In the UV-vis spectrum of the filtrate, the characteristic absorption band at 668 nm for MB disappears while the absorption band at 515 nm for EY still maintains clearly after the first run of filtration, revealing that EY passes through the membrane while MB is blocked. Similarly, the separation of other ionic dyes with opposite charges, MB@FS and MB@CR, figures out similar nanofiltration behavior (Supplementary Fig. 39). All these nanofiltrations show a high rejection efficiency of over 99.9% for cationic dyes. It should be noted that the anionic dyes do not pass through the membrane in one time, possibly because the weak hydrophobic interaction and/or the aggregation of the dyes themselves in aqueous solutions delays the permeation process. Nevertheless, after washing the residue on the membrane twice in a row, the permeation efficiency of anionic dyes reaches more than 95.9%.

Such a charge-selective separation is then extended to those surface-modified nanoparticle systems. Glutathione (GSH) modified Au NPs (Au-GSH) are prepared with an average diameter of around 2.0 nm. The freshly prepared Au NPs have a negative potential (Au−), and they pass through the membrane with high permeability in one time, as confirmed by UV-vis spectra and TEM images (Fig. 5g–i). Tuning the pH of the aqueous solution leads to the change of surface potential of Au-GSH, due to the different ionization behavior of GSH on the surface of Au NPs. Since the amino group of GSH becomes protonated under acidic conditions, the surface potential of the modified Au NPs changes to positive (Au+) while maintaining the same particle size at a low pH. As a result, the filtration experiment shows a completely opposite result, and almost all Au-GSH NPs are rejected (Fig. 5j–l). Notably, the size of all dyes (Supplementary Fig. 40, Supplementary Table 4) and Au NPs used in these separations is smaller than the aperture of the SF membrane. Therefore, the separation performance for small nanoparticles reveals that the SF membranes are applicable for not only size discrimination but also charge selective separation of molecules and nanoparticles via a simple filtration procedure.

### Charge and size-dependent separation of proteins

**Fig. 6:**
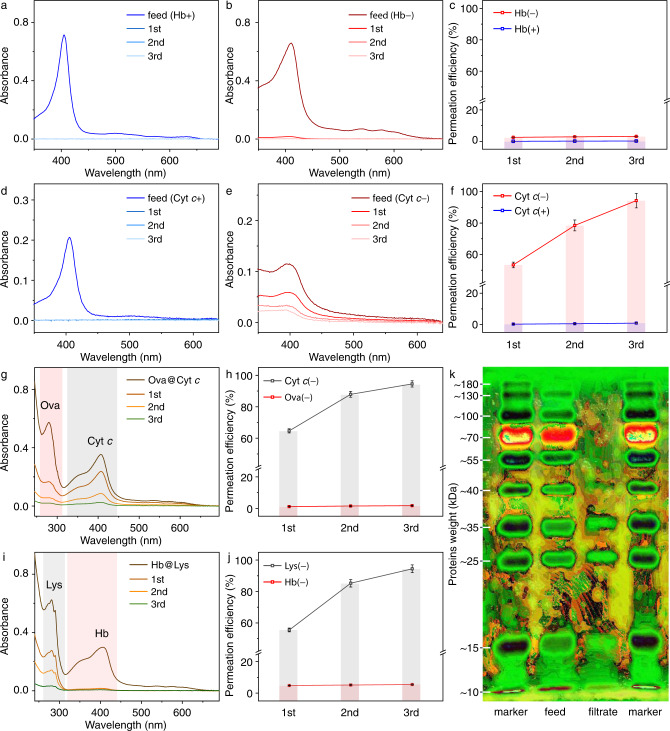
Separation of series proteins. UV-vis spectra of **a** positive and **b** negative Hb before and after filtration, and **c** permeation efficiency of Hb in the repeated separation. UV-vis spectra of **d** positive and **e** negative Cyt *c* before and after filtration, and **f** permeation efficiency of Cyt *c* in the repeated separation cycles. **g** UV-vis spectra and **h** permeation efficiency of Ova@Cyt *c* mixture in the repeated separation. **i** UV-vis spectra and **j** permeation efficiency of Hb@Lys mixture in the repeated separation cycles. Data are collected to get average values (*n* = 3). **k** SDS-PAGE analysis of the protein marker and filtrate.

The multiple intra- and inter-molecular hydrogen bonds of peptides make proteins possess definite morphologies and nanoscale sizes. In addition to the neutral state, proteins carry positive or negative charges at residue amino groups or carboxylic acid groups under different pH conditions. When the pH of the solution is above the isoelectric point (pI), the protein becomes negatively charged; below the pI, the protein turns positively charged. In principle, the membrane separation and purification for proteins are normally realized based on these properties, in which the time-saving process is important in the development and application of proteomics^62^. Since the amino acid residues surrounding the proteins framework are similar to those in Au-GSH, the SF membrane is expected to achieve the size- and charge-dependent separation of proteins. For this purpose, several regular proteins (Supplementary Fig. 41, Supplementary Table 4) are selected to identify the separation activity of the SF membrane. Hemoglobin (Hb, bovine, 5.47 × 5.72 × 6.46 nm^3^) that possesses positive surface potential in deionized water is selected to test the rejection performance of the large-size proteins (Fig. 6a–c). After filtration, the characteristic peak of Hb at 406 nm disappears basically in the filtrate, indicating effective rejection with an average rejection efficiency of 99.8%. By adjusting the pH of solutions above the pI, the SF membrane also affords an efficient rejection of 96.9% for negatively charged Hb through size discrimination, because the three-dimensional (3D) size of Hb is too large to thread the nanopores of SF. The result reveals that size screening plays a decisive role in the rejection process. Similar rejection performance of the SF membrane is found in the size screening filtration for the other two large proteins, ovalbumin (Ova, chicken, 5.64 × 8.11 × 6.84 nm^3^) and bovine serum albumin (BSA, 5.48 × 7.59 × 14.86 nm^3^), no matter what they are at negatively or positively charged state (Supplementary Fig. 42). These experiments further prove the broad applicability of the SF membrane for the size exclusion of large-size proteins. Oppositely, cytochrome *c* (Cyt *c*, bovine, 3.86 × 3.10 × 2 .31 nm^3^) in size scale smaller than the detected pores of SF assembly in two directions at the negatively charged state passes through the SF membrane readily, indicating that the membrane has no retention effect for small-size proteins (Fig. 6d–f). After washing twice in a row, 94.3% of Cyt *c* permeates the membrane, similar to the separation process of anionic dyes but performing a smaller flux due to the decreased molecular diffusion rate and the complicated Brownian dynamics of proteins^63–65^. However, when Cyt *c* is adjusted to a positive surface potential state, it is blocked with a very high rejection efficiency of 99.6%, due to the strong electrostatic interaction between the charged proteins and membrane^66^. The SF membrane displays an analogous performance for the protein lysozyme (Lys, chicken, 3.24 × 2.86 × 4.27 nm^3^, Supplementary Fig. 43). Notably, the circular dichroism spectrum of the proteins in the filtrate shows the same Cotton signals as the feed (Supplementary Fig. 44), confirming the retained protein structure^67^. These separation tests demonstrate the uniformity of porous structure in SF membrane and the corresponding applicability for purification and separation of normal proteins via both size and charge selectivity.

Besides the separation of mono-component proteins, a selected protein mixture of Ova and Cyt *c*, which both are in the negatively charged state, is used to evaluate the separation performance of the SF membrane (Fig. 6g, h). The bands at 281 and 408 nm in the UV-vis spectra are assigned to the characteristic absorptions of Ova and Cyt *c*, respectively. After filtration, the absorption at 281 nm almost disappears, and the absorption at 408 nm remains in the filtrate, demonstrating that most Ova proteins have been isolated from Cyt *c*. Significantly, after encountering twice washing procedures, ca. 94.5% of Cyt *c* passes through the membrane. In addition, the protein mixture of Hb and Lys at their negative surface potential state is used to conduct the same separation procedure (Fig. 6i, j). About 94.6% of Hb is rejected while 94.5% of Lys permeates the membrane during the filtration process. As a further extension of the separation experiment, a similar treatment to the protein mixture of BSA and Cyt *c* allows trapping 90.8% of BSA while 91.0% of Cyt *c* permeates through the membrane (Supplementary Fig. 45), showing the commonality of the SF nanosheet assembly in proteins separation. Interestingly, the membrane shows a high selectivity factor of 55 for the mixture of Ova and Cyt *c* (Supplementary Fig. 46) and is significantly higher than the separations of the other two groups. The mechanism of specific selectivity is unclear at the present stage due to the surface complexity and structural flexibility of the proteins in the separation process.

To figure out the precise separation window of the SF membrane in sieving proteins, a commercial protein marker, which contains a prestained mixture of ten recombinant proteins with molecular weights ranging from 10 to 180 kDa, is used in the filtration experiment of a multi-components system (Fig. 6k). Because the proteins in the commercial protein marker are denaturation and show negative charges, the separation is completely treated to follow a process of size screening, which is related to the molecular weight of the proteins. After simple filtration, the filtrate is used in the analysis of sodium dodecyl sulfate-polyacrylamide gel electrophoresis (SDS-PAGE). By taking the original protein marker as the reference, the SDS-PAGE experiment shows that only those proteins with molecular weight (MW) lower than 35 kDa are observed in the filtrate while the other proteins with a molecular weight higher than 40 kDa are rejected. Thus, the result proves that the critical value of molecular weight to be separated falls definitely between 35 and 40 kDa and the separation precision is smaller than 5 kDa. This accuracy of separation further demonstrates the unique separation performance of protein mixtures compared with commercial dialysis membranes, which usually require at least about tenfold molecular weight difference of proteins recommended by manufacturers^68^.

### Sustainability of SF membrane in protein separation

To further investigate the tolerance of the SF membranes against fouling in protein separation, 5 days of continuous and interrupted separation experiments of Ova ([Ova] = 1 × 10^−4^ mg/L) is conducted (Supplementary Fig. 47). The rejection efficiency of Ova remains almost unchanged in the first 40 h of the continuous separation experiments, but it gradually raises after that and comes to 99.5% on the last day. The flux remains at a constant level approximately during the first three days of continuous separation, but it shows a downward trend in the subsequent intermittent separation experiments and abates by 20.0% in total on the 5th day, which may be the result of the continuous stacking of Ova proteins under the vacuum filtration process. However, the smooth separation suggests a sustained anti-fouling capability for the SF membrane within about 3 days.

The static adsorption experiment is carried out to evaluate the adsorption capacity of the SF assemblies quantitatively, which shows that SF membrane entraps proteins with positive charges in a high adsorption quantity but the adsorbing capacity is relatively lower than the negatively charged proteins (Supplementary Fig. 48). Based on the results, the electrostatic interaction between proteins and SF assemblies can be considered the prominent factor in the evaluation of the adsorption behavior of proteins. Thus, by turning the pH of the treated solution over pI, the trapped proteins appear negatively charged and are released from the SF assemblies under a sonication. The refreshed SF assemblies collected by centrifugation are then reused to prepare the next membrane, and both permeability and rejection efficiency exhibit durative performance even after 50 cycles (Supplementary Fig. 49), revealing the well-recyclable utilization of the SF assemblies. Importantly, in the aqueous solutions with pH 1–12, PCT shows enduring stability for at least an overnight period and the host-guest interaction is affected very little. Therefore, the stability of the SF membrane sustains satisfaction in a basic or acidic environment. As indirect evidence, the filtration experiment of Cyt *c* and Ova suffering various pH conditions confirms the repeatable behavior of separation (Supplementary Fig. 50). When both Cyt *c* and Ova are in the positive charge state, the rejection efficiency of both proteins is over 99.4% due to the electrostatic adsorption. With increasing pH of the protein solution, the Cyt *c* is still positively charged while Ova comes to a negatively charged state, yet the interception of the SF membrane for both proteins does not change because of either size sieving (to Ova or the aggregates of Ova and Cyt *c*) or electrostatic adsorption (to Cyt *c*). When both Cyt *c* and Ova are in the negative charge state under an increased pH, only Ova is repelled but Cyt *c* passes through the membrane based on the size-dependent separation. All these acid/base involved experiments are recycled five times and no obvious difference in flux and efficiency is detected, suggesting that the SF membrane preserves separation properties against both basic and acidic solutions.

Considering some proteins are stabilized only in salt solutions, the separation performance under different salt concentrations is evaluated. The results appear that both flux and efficiency are not affected even if the concentration of salt is raised to about 20-fold of normal saline (Supplementary Fig. 51) because the salt has almost no effect on the solubility of the framework assembly and the host-guest interaction. Interestingly, increasing the area of the separation membrane by four times (~12.56 cm^2^) does not affect the rejection efficiency and flux evidently (Supplementary Fig. 52), confirming that the SF membrane can be scaled up without obvious defects. Furthermore, a dosage-dependent separation (Supplementary Fig. 53) shows that even after 10^4^ mL feed solution ([Ova] = 1 × 10^−4^ mg/L) is used at one time, the SF membrane still provides an effective rejection for proteins with an efficiency of 99.5% though the flux is reduced by 14.2%.

## Discussion

Following the initial motivation, we succeed in the fabrication of a novel processible 2D SF structure that bears inner layer hexagonal pores via the 3 + 2 types of host-guest inclusion of a linear inorganic cluster PCT bearing methoxypillar[5]arene groups at both ends with a trident organic guest. Furthermore, the 2D SFs in the form of a layered assembly are exfoliated by adjusting the polarity of the solvent and further processed into membranes by a sample suction under reduced pressure. In contrast to those crystalline frameworks based on coordination or covalent bonding, the intrinsic structural flexibility of the SF sourcing from host-guest inclusion and the large size of the layer ensures the tight packing of layered SF during the filtration so that the formed SF membrane has uniformed and high-density nanopores without detectable staking defects. Importantly, the polyanionic POM cluster inserted in the skeleton of the framework plays an indispensable role in bracing the framework and providing charged surface environment. These structural advantages of SF assembly have been confirmed to be valuable in the functional membrane separation processes. The strict size- and charge-dependent sieving of Au NPs, dyes, and even proteins are realized through a convenient filtration with a cut-off value of 3.8 nm in size scale and the separation precision smaller than 5 kDa in sieving the mixture of proteins, which allows the separation performance of SF membranes to be applicable at the forefront (Supplementary Tables 5–7). At the present stage for the membrane comprising randomly spread 2D assemblies, it is still hard to make sure that the penetration channels and screening are completely derived from the framework pores based on the possibility of filtration passing through the gap between SF assemblies. As a comparison, the SF membranes mixing with polycaprolactone (PCL) at a weight ratio of 1:1 show almost the same cut-off values of ca. 4.0 nm for both filtrate and retentate in the separation of Au NPs as the membrane without PCL (Supplementary Figs. 54–56), supporting that the framework pores play a dominated role in the separation experiments. The repeated filtration experiments presenting almost the accordant separation performance verify that the SF membrane can work continuously for 3 days without a noticeable decrease in performance. Fortunately, the membrane displays anti-fouling and tolerance to the pH (1–12) and salt (20-fold of normal saline) in solutions, and the dosage enlargement (10^4^ mL). With the structural stability of SF assembly, the porous material allows the membrane to be recycled (>50) and scaled up without obvious defects. The applicability to various molecules and nanoparticles confirms that the SF membrane is suitable for a wide range of separation systems, including chemicals, nanomaterials, and biomolecules. We believe that the present breakthrough in SF membranes as an on-demand platform also offers coming opportunities for chiral separation and the combination of catalysis and separation.

## Methods

### Preparation of SF assembly

PCT (30 mg, 8.32 mmol) and TT (3.33 mg, 5.55 mmol) which are synthesized following procedures described in the Supplementary Information are dissolved in 10 mL of chloroform, respectively. They are then mixed together with a molar ratio of 3:2 (PCT:TT) to allow the formation of 2D SFs. The 2D SF assembly starts to float on the surface of the solution within 1.0 h at room temperature, which is then separated through filtration and dried after washing with CHCl_3_.

### Liquid exfoliation of SF assembly

The prepared SF assembly is dispersed in the mixed solvent (1.00 mg/mL) of DMSO/H_2_O (1:4, in v/v) and then encounters a sonication for 30 min to get a homogeneous solution.

### Preparation of SF membrane

The SF membranes for structural characterizations and separation experiments are prepared through a simple filtration procedure under gradient-reduced pressures from −0.02 to −0.08 MPa, by using the commercial porous substrates (200~450 nm, pore size) as the support. Polycarbonate, polyester, cellulose ester, polyvinylidene fluoride, polytetrafluoroethylene, and nylon membranes, etc. are all applicable. The concentration is normally set at 1.00 mg/mL based on SFs (if not specified). The thickness of the membranes is tuned via the control of filtration volumes. The freshly prepared membrane is washed with water three times to make sure that no SFs leach for the next separation experiment.

### Separation experiments and calculation

Typically, the series separation experiments are carried out under a reduced pressure of −0.08 MPa. For the filtration procedures of anionic dyes and proteins, a repeated washing step (three times in most situations) is adopted to make most molecules pass through the membranes.

For one separation, the rejection efficiency (${\eta }_{R}$) is determined by:

1 $${\eta }_{R}=\left(1-\frac{{C}_{p}}{{C}_{f}}\right)\times 100\%$$

where ${C}_{f}$ and ${C}_{p}$ represent the concentrations of feed and permeate solutions, which are determined by the absorbance in UV-vis spectra according to the working plot. At least three parallel measurements are carried out to get the arithmetic average values.

For repeated separations, the permeation efficiency (${\eta }_{P}$) is calculated from the following equation:

2 $${\eta }_{P}=\left(\frac{\sum {I}_{n}{V}_{n}}{{I}_{0}{V}_{0}}\right)\times 100\%$$

where ${I}_{n}$ and ${V}_{n}$ denote the absorbance and volume of the permeated solution after *n* times of separation, ${I}_{0}$ and ${V}_{0}$ represent the absorbance and volume of the feed. At least three parallel experiments are carried out to get average values.

The separation factor is calculated from:

3 $${\alpha }_{i/j}=\frac{{\left(\frac{{C}_{p}}{{C}_{f}}\right)}_{i}}{{\left(\frac{{C}_{p}}{{C}_{f}}\right)}_{j}}=\frac{{\eta }_{{pi}}}{{\eta }_{{pj}}}=\frac{{\left(\frac{\sum {I}_{n}{V}_{n}}{{I}_{0}{V}_{0}}\right)}_{i}}{{\left(\frac{\sum {I}_{n}{V}_{n}}{{I}_{0}{V}_{0}}\right)}_{j}}$$

where ${C}_{f}$ and ${C}_{p}$ represent the concentrations of feed and permeate, and the concentrations are obtained from the relevant UV-vis spectra according to the working plot. ${I}_{n}$ and ${V}_{n}$ are the absorbance and volume of permeated solution after *n* times of separation, ${I}_{0}$ and ${V}_{0}$ afford the absorbance and volume of the feed solution, and $i$ and $j$ denote the components in the mixed solution. At least three parallel experiments are carried out.

The flux ($J$) under −0.08 MPa at room temperature is calculated by:

4 $$J=\frac{V}{(t\times A)}$$

where $V$ is the filtrate volume (*L*), $A$ is the effective filtration membrane area (in m^2^), and $t$ affords the separation time (h). The data are collected over three parallel experiments.

### Static adsorption experiment

The prepared SF assembly is dispersed in protein solutions with a concentration of 1 mg/mL, after treatment with sonication for 30 minutes, the solutions are allowed to stay at 4 °C overnight. The adsorption capacity (*Γ*) is calculated by:

5 $$\varGamma=\frac{\frac{{I}_{0}-I}{{I}_{0}}\times {m}_{0}}{{m}_{{SF}}}$$

where ${I}_{0}$ and $I$ are the absorbance of proteins before and after the adsorption process, respectively, ${m}_{0}$ is the mass of proteins (mg) before adsorption, and ${m}_{{SF}}$ is the mass of SF assembly (g). The data are collected over three parallel experiments.

### SDS-PAGE experiment

The SDS-PAGE analysis is performed as reported for both feed and filtrate solutions to examine the molecular weight cut-off of the SF membrane. Typically, a 12% sodium dodecyl sulfate-polyacrylamide gel is used as the separation gel plate, and the samples are used without further treatment before the electrophoresis. The voltages of stacking gel are set as 80 and 120 V for separation gel. After completing the electrophoresis, the gel is dyed with Coomassie blue for 1 h and then faded to get the clear protein bands.

## Supplementary information

Supplementary Information

Description of Additional Supplementary Files

Supplementary Movie 1

Supplementary Movie 2

Supplementary Movie 3

Supplementary Movie 4

## Data Availability

The raw data generated in this study are provided in the Source Data file. Source data are provided in this paper.

## Source data

Source Data
