# Supplementary material for: Supramolecular framework membrane for precise sieving of small molecules, nanoparticles and proteins
Source: Nat Commun. 2023 Feb 22;14:975. doi: 10.1038/s41467-023-36684-w (PMC9944550; doi:10.1038/s41467-023-36684-w)
Supplement: Supplementary file 1 — Supplementary Information [file 41467_2023_36684_MOESM1_ESM.pdf]

Supplementary Information

Supramolecular framework membrane for precise sieving small molecules, nanoparticles and proteins

Zhang et al.

Materials

The general chemicals, N,N-diisopropylethylamine (DIPEA), 1,2-dibromoethane, 1,4-dimethoxybenzene, paraformaldehyde, 5-bromopentanenitrile and boron fluoride ethyl ether [$BF_3 \cdot O(C_2H_5)_2$] are the products of J&K Scientific Ltd. 4-methoxyphenol, 4-cyanophenol and the dyes used in the experiments were purchased from Energy Chemical Ltd. Trifluoromethanesulfonic acid and solvents were purchased from Sinopharm Chemical Reagent Co., Ltd. Ovalbumin (chicken), cytochrome *c* (bovine), and hemoglobin (bovine) were received from Sigma-Aldrich Co. LLC. Lysozyme (chicken), and bovine serum albumin are the products of Shanghai Aladdin Biochemical Technology Co., Ltd. All the compounds were used without further purification. The PageRuler™ Prestained Protein Ladder with a prestained mixture of ten recombinant proteins ranging from 10 kDa to 180 kDa was purchased from ThermoFisher Scientific Co., Ltd. All the solvents were analytical grade and used as received except that 1,2-dichloroethane (DCE) was dried with activated molecular sieves (4A) for days and distilled just before using. Doubly distilled water was used in the experiments. Silica gel (300−400 mesh) was applied for column chromatography.

Measurements

^{1}H NMR and 2D NOESY NMR spectra were recorded on a Bruker Avance 500 MHz spectrometer (Germany) by using tetramethylsilane (TMS) as internal reference (s= singlet, br= broad, d =doublet, t= triplet, q= quartet, m= multiplet). FT-IR spectra (KBr pellet) were collected on a Bruker Vertex 80 V spectrometer (Germany) equipped with a DTGS detector (32 scans) in a resolution of 4 cm^{-1}. UV-vis spectra were carried out on a Varian CARY 50 Probe spectrometer (USA). Organic elemental analyses (C, H, N) were carried out on a Vario micro cube (Elementar, Germany). MALDI-TOF mass spectra were recorded on a matrix assisted laser desorption ionization (MALDI) time of flight (TOF) mass spectrometer (Bruker Autoflex^{TM} speed TOF/TOF, Germany), equipped with a nitrogen laser (337 nm, 3 ns pulse). The matrix was trans-2-[3-(4-tert-Butylphenyl)-2-methyl-2-propenylidene]malononitrile (DCTB). The mass to charge ratio range during datum acquisition is from 700 to 2000 Da for reflection positive mode and 5K to 20K Da for linear positive mode. LC-ESI mass spectra were carried out by POEMS inductively coupled plasma mass spectrometer (TJA, USA). GC mass spectra were carried out by Ion trap gas chromatography (GC) mass spectrometer (Thermo Fisher-ITQ1100, USA) with electron impact (EI) ionization mode. TEM images were obtained on a field emission transmission electron microscope (JEOL JEM-2100F, Japan) with accelerating voltage of 200 KV without staining. SEM measurements were performed on a JEOL JSM-6700F (Japan) field emission scanning electron microscope. AFM images were taken with a Dimension FastScanTM atomic force microscope (Bruker, USA) under ambient conditions. X-ray diffraction (XRD) data were recorded on a Rigaku SmartLab 3 (Japan) X-ray diffractometer and a Bruker D8 Venture X-ray diffraction using Cu Kα1 radiation at a wavelength of 1.542 Å. ITC data were obtained by using MicroCal VP-isothermal titration calorimeter (Malvern, UK). N_2 sorption experiment was carried out on a Beishide instruments 3H-2000PS2 (China). Zeta potential measurements were performed by using a Malvern Zetasizer NanoZS (UK) instrument at room temperature. Circular dichroism (CD) spectra were performed on a Bio-Logic MOS-450 spectropolarimeter with step size of 0.5 nm and speed of 0.5 nm s^{-1} at 25 °C.

Synthesis of PCT host and TT and MT guests

Aminoethyl pillar[5]arene (AP)[1]: The synthesis of AP is followed the route shown in Supplementary Fig. 1. To a solution of BB (2.77 g, 12.00 mmol) that is prepared from the etherification of 4-methoxylphenol and 1,2-dibromoethane, 1,4-dimethoxybenzene (6.63 g, 48.00 mmol) and paraformaldehyde (1.82 g, 60.00 mmol) in anhydrous 1,2-dichloroethane (200 mL), were added under N_2 atmosphere and stirred for 10 minutes. Then, boron fluoride ethyl ether ([$BF_3 \cdot O(C_2H_5)_2$]) (7.50 mL, 60.00 mmol) was added. The mixture solution was stirred under N_2 atmosphere at 28°C for another 3.5 h. A mixture of water and methanol (1:1, v/v) was added and stirred for 2 h. The product was extracted with CH_2Cl_2 after filtration, the organic phase was concentrated and dried over Na_2SO_4. After evaporation of the solvent under reduced pressure, the residue was purified by column chromatography using CH_2Cl_2 as eluent to afford crude product bromine methoxy pillar[5]arene (BP) for the next step reaction. The incompletely purified BP (5.11 g, 6.06 mmol), phthalimide (1.27 g, 8.66 mmol), and K_2CO_3 (1.34 g, 9.69 mmol), were dissolved in DMF at room temperature, and the solution was heated up to 60°C with stirring overnight under N_2 protection. After cooling to room temperature, the reaction mixture was filtered and the solvent was removed under reduced pressure to give a crude product, which was used in the next step directly. The crude solid was dissolved in THF (60 mL) at 65 °C and $NH_2NH_2 \cdot H_2O$ (30 mL, 612 mmol) was added, the reaction was carried out

overnight. After finishing the reaction, the solvent was removed. Saturated sodium carbonate was added and extracted with dichloromethane (30 mL×3). The organic phase was collected and dried with anhydrous sodium sulfate. Further purification was carried out by column chromatography using CH_2Cl_2: MeOH (40:1, v/v) as eluent to afford 1.62 g (2.01 mmol) of product. Yield for total three steps: 16.7%. ^{1}H NMR ($CDCl_3$, 500 MHz, 25°C): δ 6.77−6.66 (m, 10H), 3.80−3.78 (br, 2H), 3.78−3.74(m, 10H), 3.68−3.57(m, 27H), 2.88−2.84 (t, 2H), as shown in Supplementary Fig. 2. Elemental analysis for $C_{46}H_{53}O_{10}N$ (779.91 g/mol): C, 70.84%; H, 6.85%; N, 1.80%, found: C, 70.88%; H, 6.65%; N, 1.81%. GC-MS (m/z) $[M]^+$: calculated for $C_{46}H_{54}O_{10}N$: 780.92, found: 780.44, as shown in Supplementary Fig. 3.

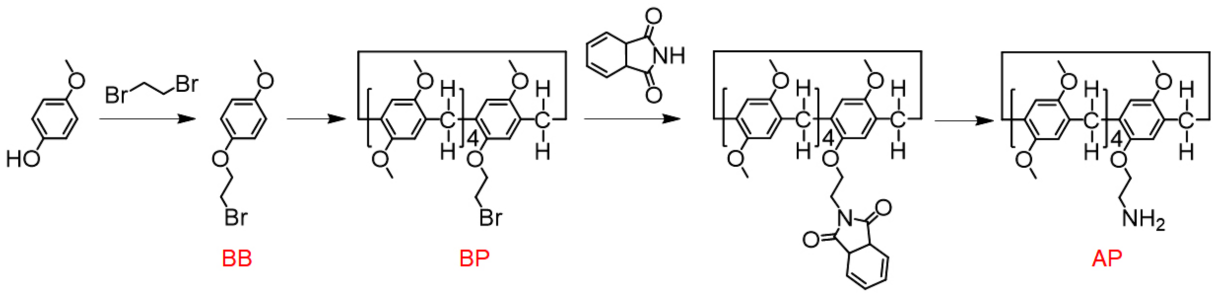

Supplementary Fig. 1 Synthetic route. AP.

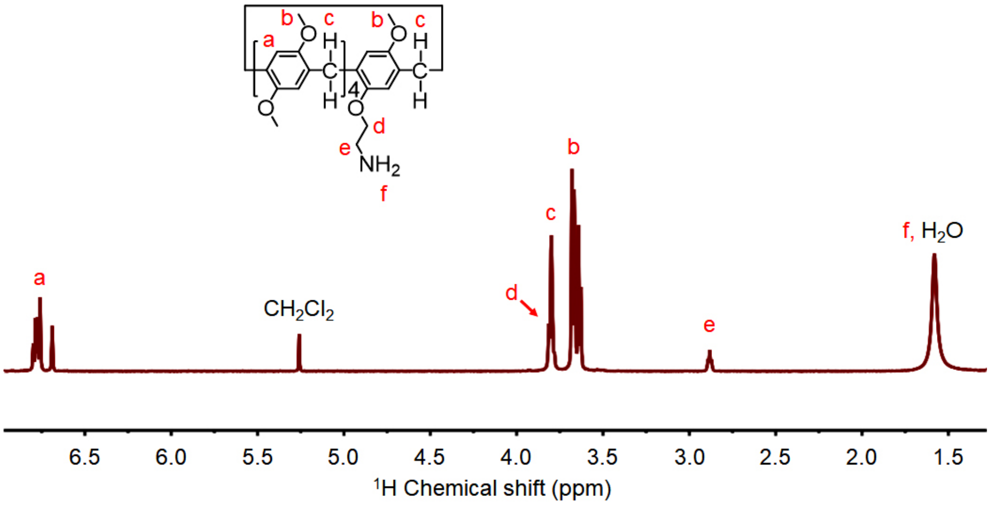

Supplementary Fig. 2 ^{1}H NMR spectrum. AP in $CDCl_3$ (500 MHz, 25°C).

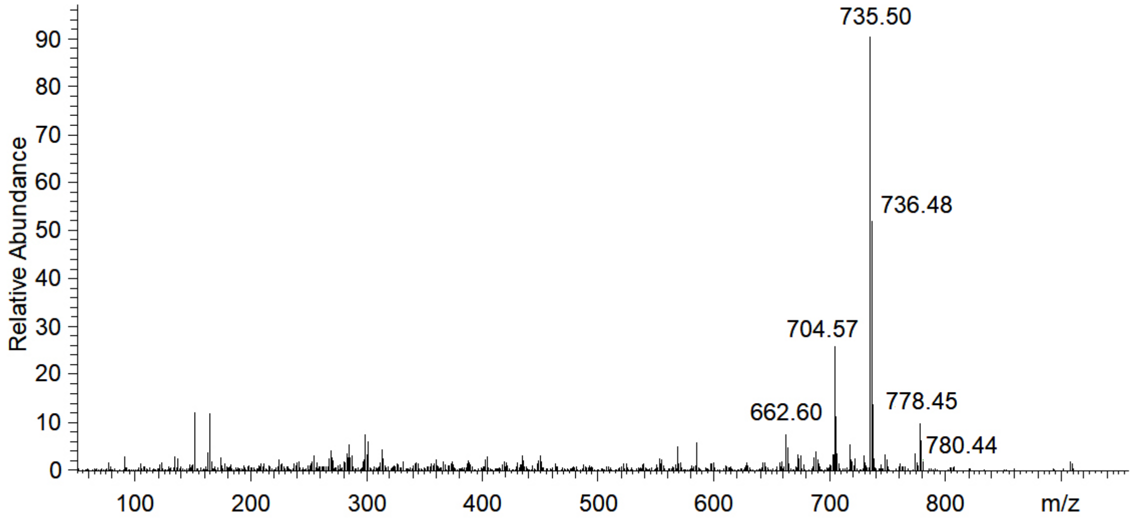

Supplementary Fig. 3 GC-MS. AP.

PCT: The synthesis of PCT is followed the route shown in Supplementary Fig. 4. A mixture of AP (0.58 g, 0.75 mmol), NHS-$MnMo_6$[2] (0.88 g, 0.37 mmol), and N,N-Diisopropylethylamine (DIPEA, 0.88 mL, 4.75 mmol) was dissolved in DMF (30 mL), and the solution was stirred vigorously for 24 h at room temperature. The excess solvent was removed by rotary evaporation under vacuum. The crude product was dissolved in $CHCl_3$ and then underwent a filtration to collect the filtrate. The ethyl acetate was added to the filtrate and the formed precipitate was collected, giving the target product (1.06 g, 0.29 mmol). Yield (78.4%). ^{1}H NMR (DMSO-d_6, 500 MHz, 25°C): δ 66.41−61.32 (br, 12H), 8.20−8.10 (br, 2H), 7.84−7.12 (br, 2H), 6.89−6.70 (m, 20H), 3.88−3.80 (br, 4H), 3.79−3.62 (m, 74H), 3.52−3.46 (br, 4H), 3.22−3.12 (t, 24H), 2.77−2.66 (br, 4H), 2.36−2.26 (br, 4H), 1.63−1.52 (m, 24H), 1.37−1.27 (m, 24H), 0.99−0.90 (t, 36H), as shown in Supplementary Fig. 5. Elemental analysis for $(C_{16}H_{36}N)_3(C_{54}H_{63}O_{12}N_2)_2MnMo_6O_{24}$ (3606.12 g/mol): C, 51.96%; H, 6.54%; N, 2.72%, found: C, 51.68%; H, 6.49%; N, 2.68%. ^{13}C NMR (DMSO-d_6, 500 MHz, 25°C): δ 173.52, 171.99, 150.40, 149.59, 128.25−127.87, 114.74, 114.30, 113.81, 67.42, 58.04, 56.18, 56.11, 55.95, 32.14, 29.85, 29.57, 29.46, 29.30, 23.58, 19.79, 14.06, as shown in Supplementary Fig. 6. ESI-MS spectra and peak assignments were shown in Supplementary Fig. 7 and Supplementary Table 1. MALDI-TOF-MS (m/z) [M+TBA]$^+$: calculated for $[(C_{16}H_{36}N)_4(C_{54}H_{63}O_{12}N_2)_2MnMo_6O_{24}]^+$: 3848.587, found: m/z = 3848.608, as shown in Supplementary Fig. 8. FT-IR (KBr pellet): v= 3686−3134, 3103−2759, 1718−1600, 1501, 1467, 1446, 1399, 1211, 1044, 941, 919, 900, 671 cm^{-1}, as shown in Supplementary Fig. 9.

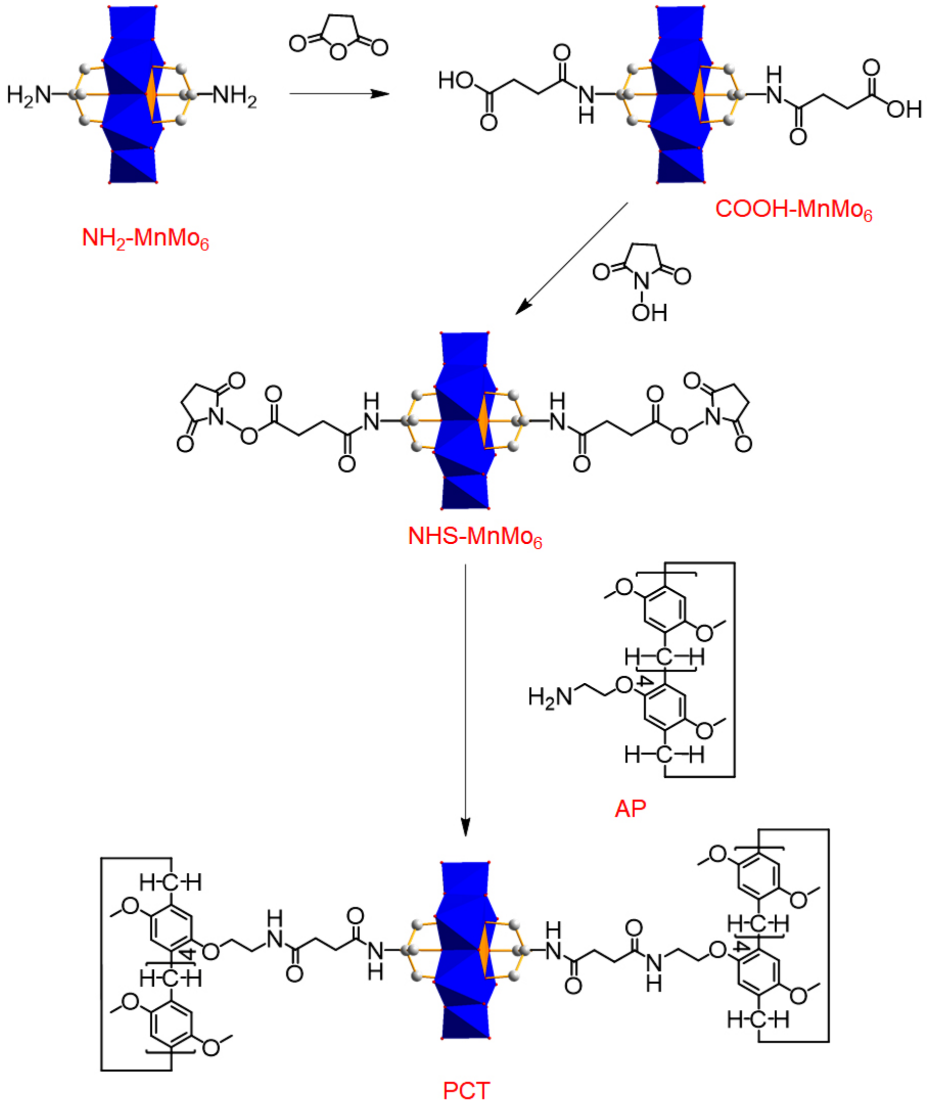

Supplementary Fig. 4 Synthetic route. Pillar[5]arene modified cluster, PCT, in which counterion TBA is omitted.

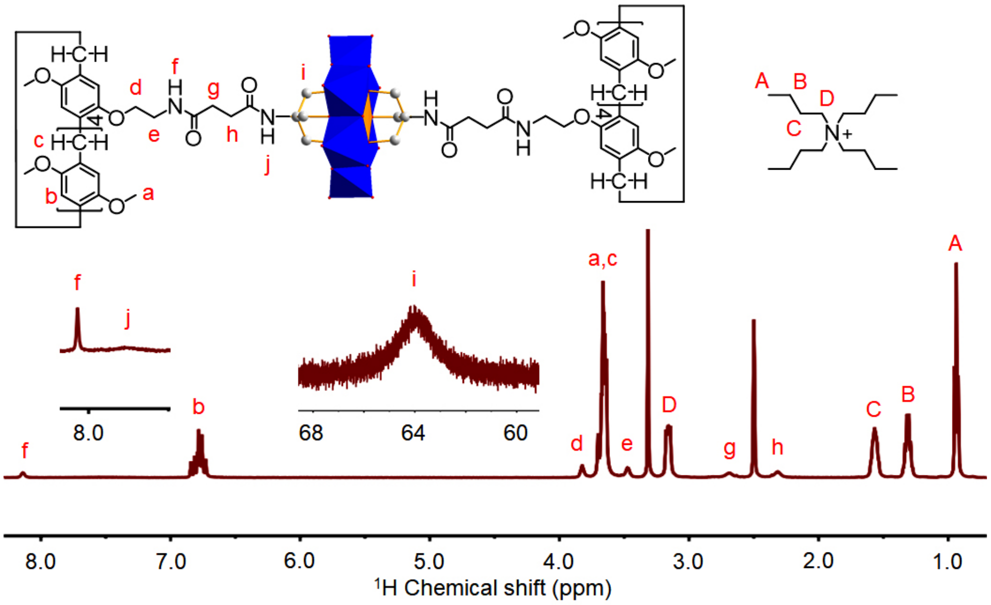

Supplementary Fig. 5 ^{1}H NMR spectrum. PCT in DMSO-*d₆* (500 MHz, 25°C).

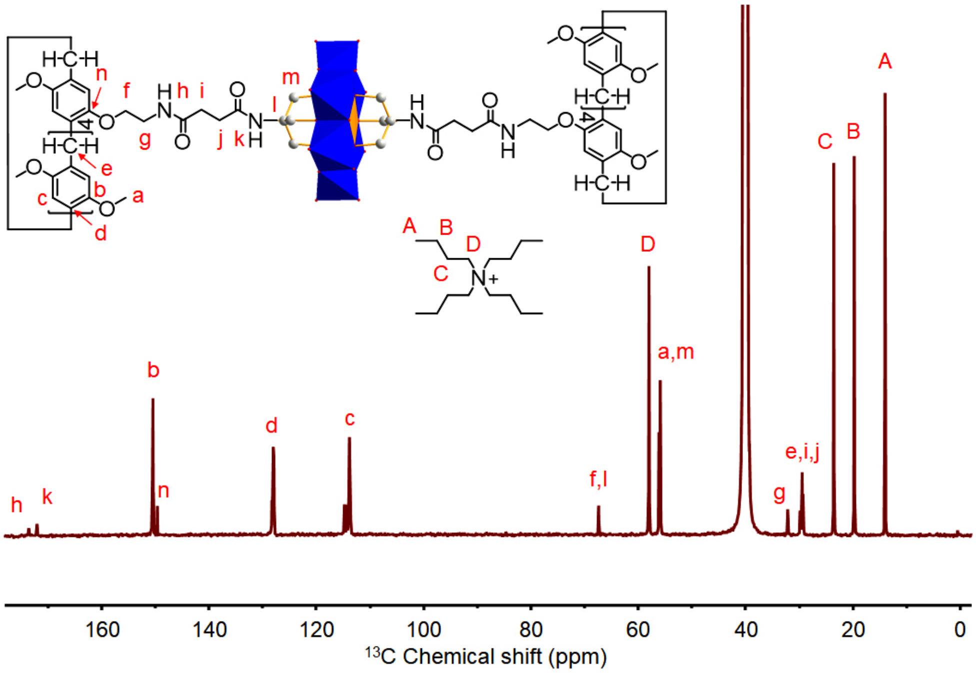

Supplementary Fig. 6 ^{13}C NMR spectrum. PCT in DMSO-*d₆* (500 MHz, 25°C).

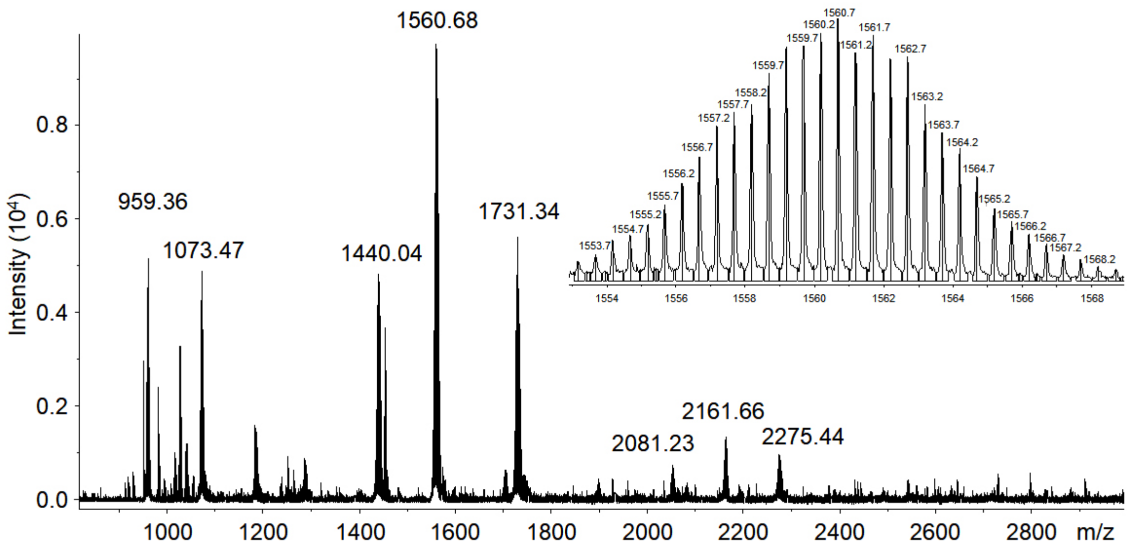

Supplementary Fig. 7 ESI-MS. PCT, where those fragment peaks are assigned in Supplementary Table 1.

Supplementary Table 1. Lists of the peak assignments found in the ESI-MS spectrum of PCT, shown in Supplementary Fig. 6. Deprotonation of the ligand is highlighted in red.

Formula assigned	z	m/z calculated	m/z observed
$[(C_{54}H_{63}O_{12}N_2)_2MnMo_6O_{24}]$	−3	959.58	959.36
$(C_{16}H_{36}N)[(C_{54}H_{63}O_{12}N_2)(C_{54}H_{62}O_{12}N_2)MnMo_6O_{24}](CH_3CN)_2(H_2O)$	−3	1073.44	1073.47
$H(C_{16}H_{36}N)_2[(C_{54}H_{63}O_{12}N_2)_2MnMo_6O_{24}]_2$	−3	2081.13	2081.23
$(C_{16}H_{36}N)_3[(C_{54}H_{63}O_{12}N_2)_2MnMo_6O_{24}]_2$	−3	2161.62	2161.66
$H(C_{16}H_{36}N)_4[(C_{54}H_{63}O_{12}N_2)(C_{54}H_{62}O_{12}N_2)MnMo_6O_{24}]_2(CH_3CN)_2(H_2O)$	−3	2275.48	2275.44
$(C_{16}H_{36}N)[(C_{54}H_{63}O_{12}N_2)_2MnMo_6O_{24}]$	−2	1560.60	1560.68
$H[(C_{54}H_{63}O_{12}N_2)_2MnMo_6O_{24}]$	−2	1439.87	1440.04
$(C_{16}H_{36}N)_2[(C_{54}H_{63}O_{12}N_2)(C_{54}H_{62}O_{12}N_2)MnMo_6O_{24}](CH_3CN)_2(H_2O)$	−2	1731.39	1731.34

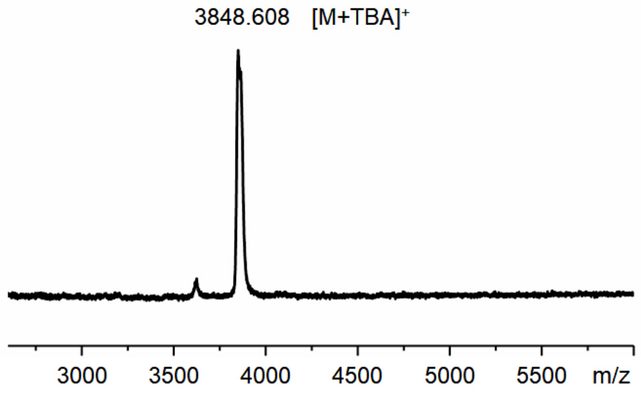

Supplementary Fig. 8 MALDI-TOF MS. PCT.

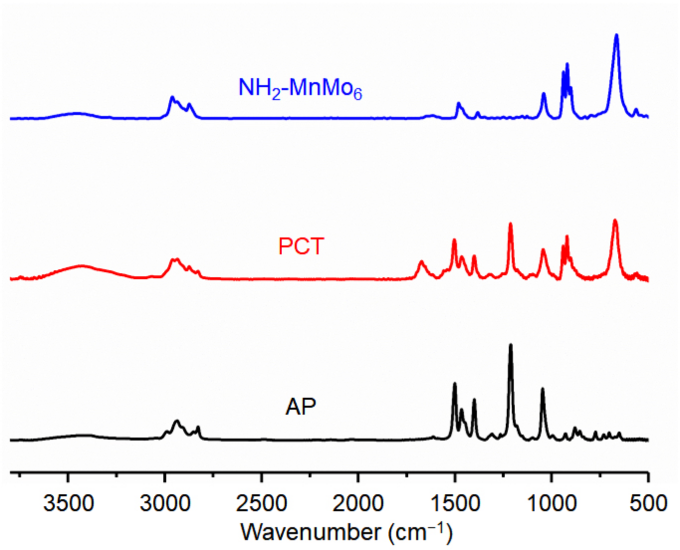

Supplementary Fig. 9 FT-IR spectra. NH_2-$MnMo_6$,[3] PCT and AP.

Tripentanenitrile-triazine (TT): The synthesis of TT is followed the route shown in Supplementary Fig. 10. 5-bromopentanenitrile (0.61 g, 3.74 mol), K_2CO_3 (0.94 g, 6.78 mmol) and triphenol-triazine[4] (0.41 g, 1.13 mmol) were added in dry acetone (50 mL) under N_2 atmosphere and refluxed overnight. After the reaction was completed, solid was filtered, the solvent was removed by evaporation under vacuum rotary evaporation. The residue was purified by column chromatography using CH_2Cl_2 as eluent to give the product as a white solid (0.33 g, 0.55 mmol). Yield 48.7%. ^{1}H NMR ($CDCl_3$, 500 MHz, 25°C): δ 8.72−8.68 (d, 6H), 7.06−7.01 (b, 6H), 4.16−4.11 (t, 6H), 2.53−2.46 (t, 6H), 2.07−1.99 (m, 6H), 1.98−1.90 (m, 6H), as shown in Supplementary Fig. 11. ^{13}C NMR (DMSO-d_6, 500 MHz, 25°C): δ 170.66, 162.25, 130.76, 129.25, 119.48, 114.28, 66.85, 28.18, 22.26, 17.07, as shown in Supplementary Fig. 12. Elemental analysis for $C_{36}H_{36}O_3N_6$ (600.71 g/mol): C, 71.98%; H, 6.04%; N, 13.99%, found: C, 72.24%; H, 6.05%; N, 13.99%. MALDI-TOF-MS (m/z) $[M+H]^+$: calculated for $C_{36}H_{36}O_3N_6H$: 601.718, found: 601.232, $[M+Na]^+$: calculated for $C_{36}H_{36}O_3N_6Na$: 623.699, found: 623.202, as shown in Supplementary Fig. 13.

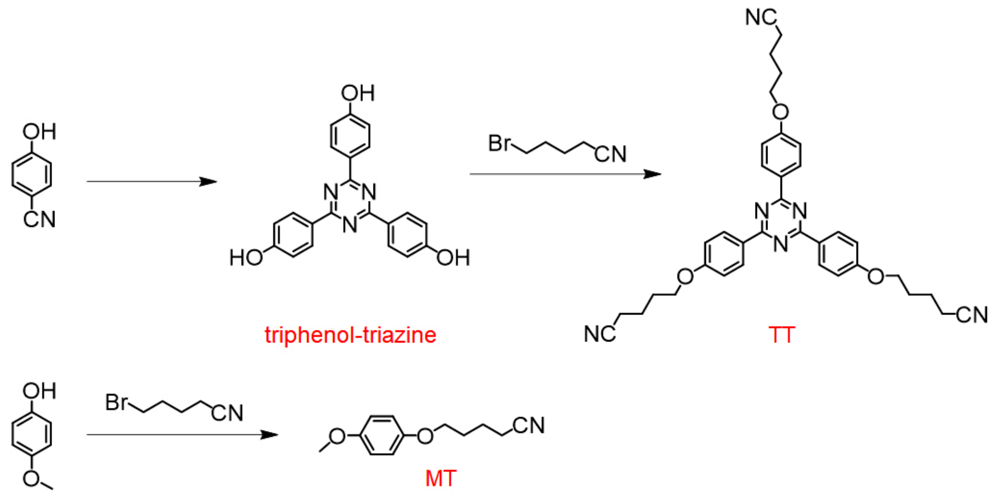

Supplementary Fig. 10 Synthetic routes. TT and MT.

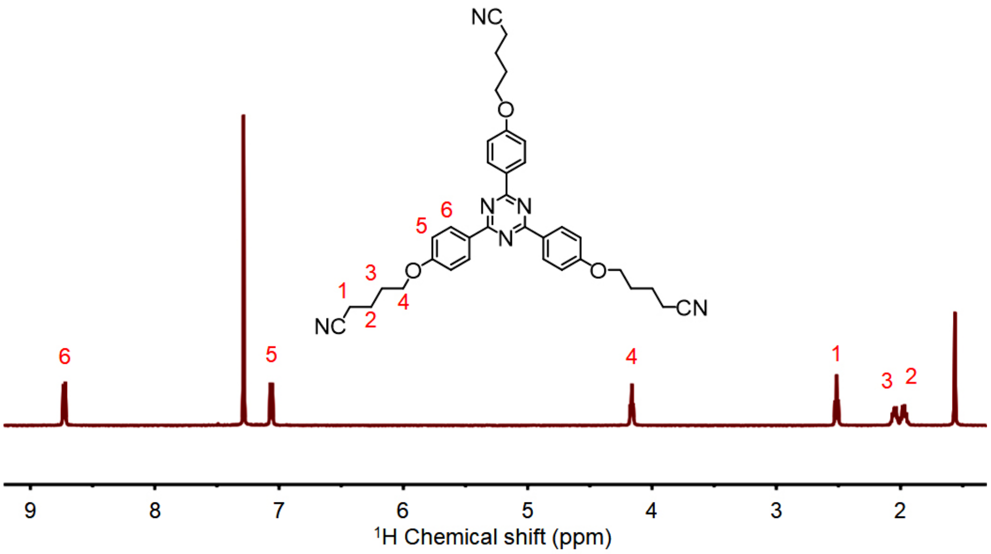

Supplementary Fig. 11 ^{1}H NMR spectrum. TT in $CDCl_3$ (500 MHz, 25°C).

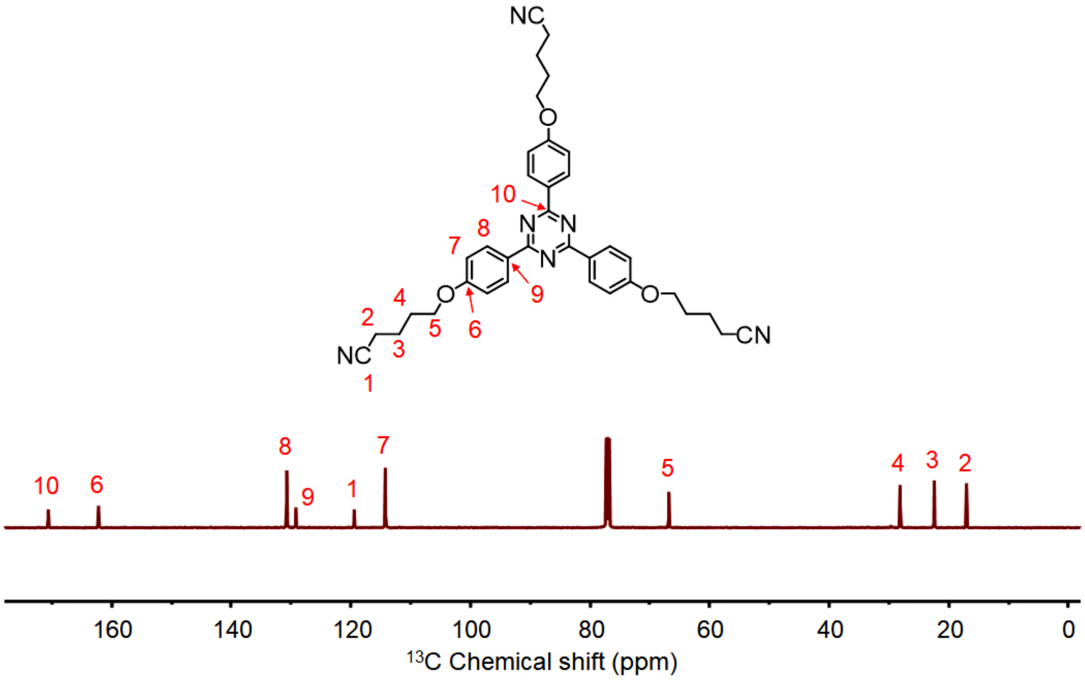

Supplementary Fig. 12 ^{13}C NMR spectrum. TT in $CDCl_3$ (500 MHz, 25°C).

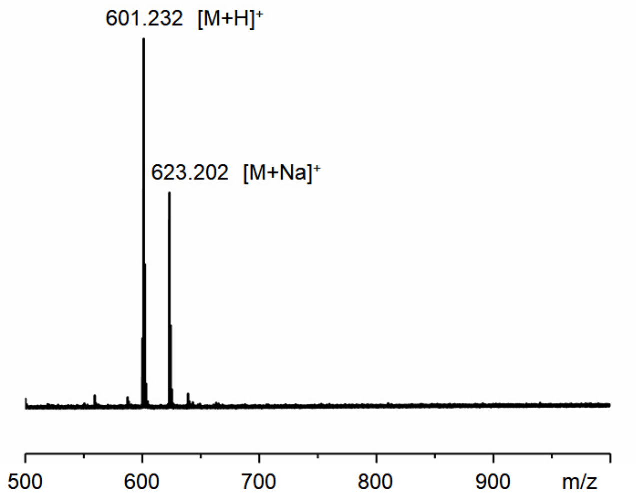

Supplementary Fig. 13 MALDI-TOF MS. TT.

4-methoxyphenoxy pentanenitrile (MT): The synthesis of MT follows the route shown in Supplementary Fig. 10. 5-bromopentanenitrile (1.56 g, 9.66 mol), K_2CO_3 (3.34 g, 24.2 mmol) and 4-methoxyphenol (1.0 g, 8.05 mmol) were added in dry acetone (50 mL) under N_2 atmosphere and refluxed overnight. After the reaction solution was cooled down, solid was filtered, the solvent was removed by evaporation under vacuum rotary evaporation. The residue was purified by column chromatography using CH_2Cl_2/n-hexane (1:1, v/v) as eluent to give the product as a white solid (1.03 g, 5.02 mmol). Yield 62.4%. ^{1}H NMR ($CDCl_3$, 500 MHz, 25°C): δ 6.88−6.83 (d, 4H), 4.00−3.96 (t, 2H), 3.79 (s, 3H), 2.49−2.44 (t, 2H), 1.99−1.87 (m, 4H), as shown in Supplementary Fig. 14. ^{13}C NMR (DMSO-d_6, 500 MHz, 25°C): δ 153.97, 152.85, 119.56, 115.40, 114.70, 67.30, 55.75, 28.30, 22.49, 17.01, as shown in Supplementary Fig. 15. Elemental analysis for $C_{12}H_{15}NO_2$ (205.25 g/mol): C, 70.22%; H, 7.37%; N, 6.82%, found: C, 70.24%; H, 7.05%; N, 6.89%. GC-MS (m/z) $[M]^+$: calculated for $C_{12}H_{16}NO_2$: 206.26, found: 205.95, as shown in Supplementary Fig. 16.

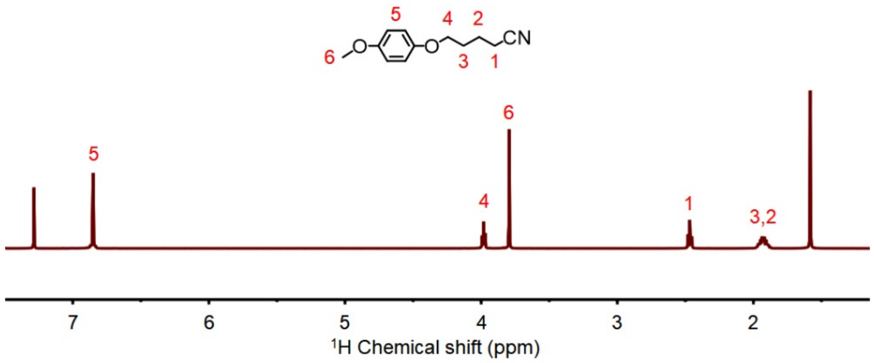

Supplementary Fig. 14 ^{1}H NMR spectrum. MT in $CDCl_3$ (500 MHz, 25°C).

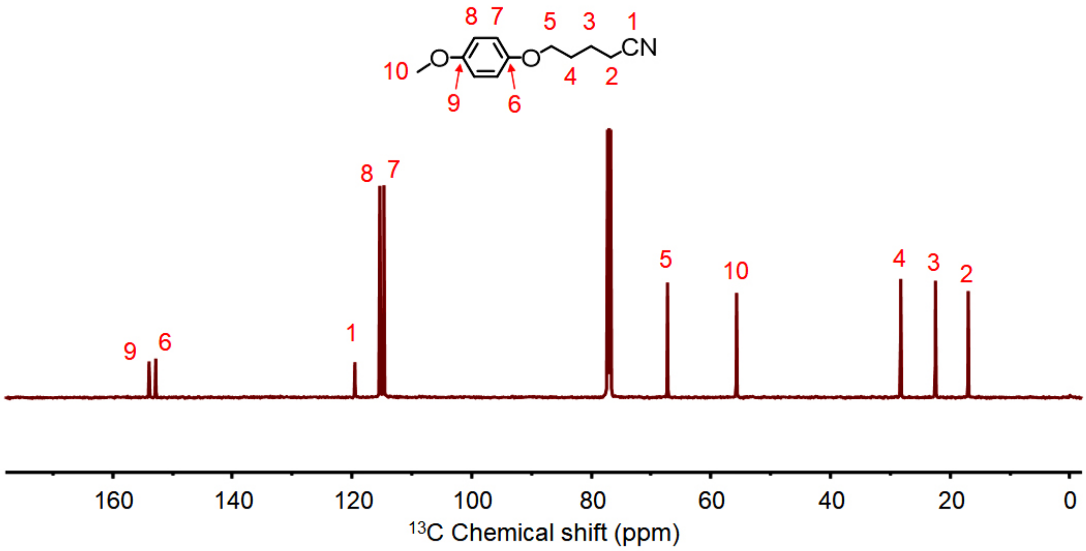

Supplementary Fig. 15 ^{13}C NMR spectrum. MT in $CDCl_3$ (500 MHz, 25°C).

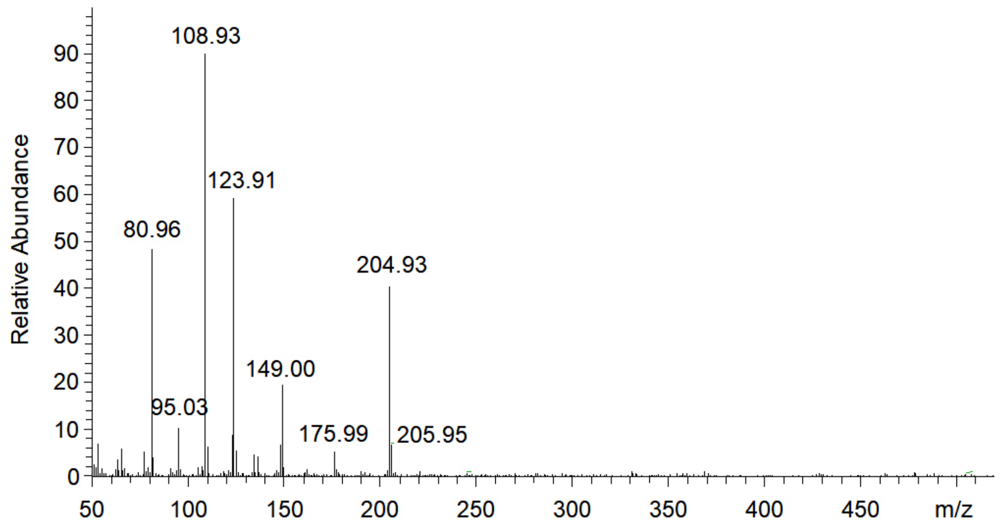

Supplementary Fig. 16 GC-MS. MT.

Host-guest interaction investigation

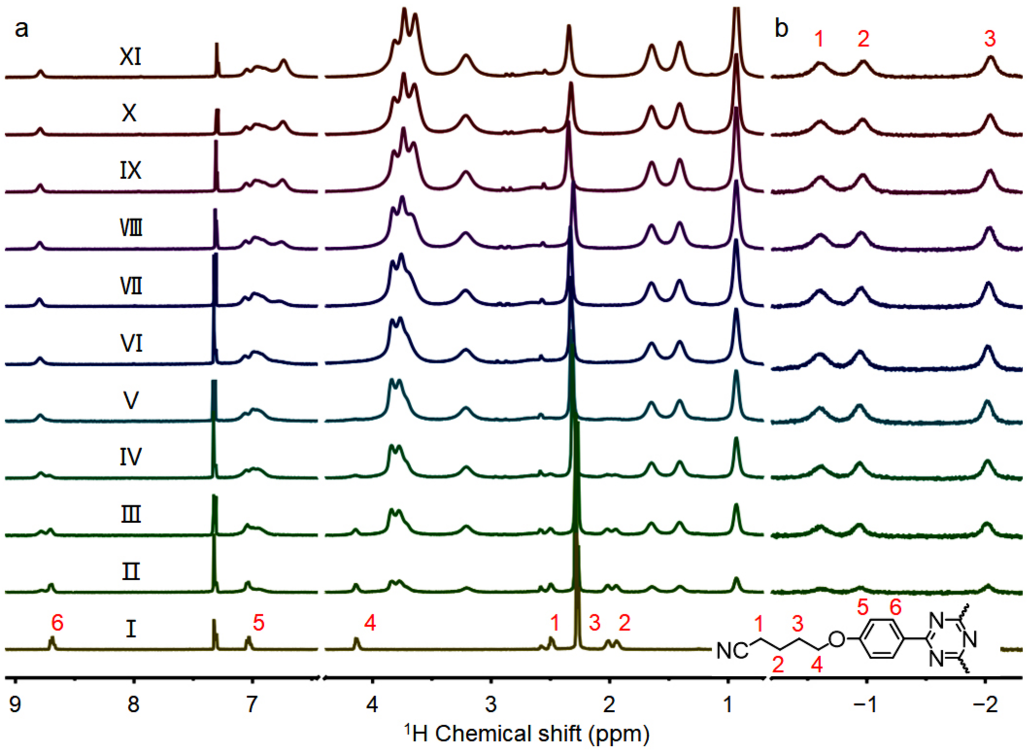

Supplementary Fig. 17 Host-guest interaction between TT and PCT. Partial ^{1}H NMR spectra ($CDCl_3$: DMSO-d_6= 50:1, 500 MHz, 25°C) of TT (2 mM) upon addition of 0 (Ⅰ), 0.3 (Ⅱ), 0.6 (Ⅲ), 0.9 (IV), 1.2 (V), 1.5 (VI), 1.8 (Ⅶ), 2.1 (Ⅷ), 2.4 (IX), 2.7 (X), 3.0 (XI) eq. PCT: **(a)** ^{1}H Chemical shift: 9.0−0.7 ppm, **(b)** Increased signals of chemical shift: 0− (−2.4) ppm.

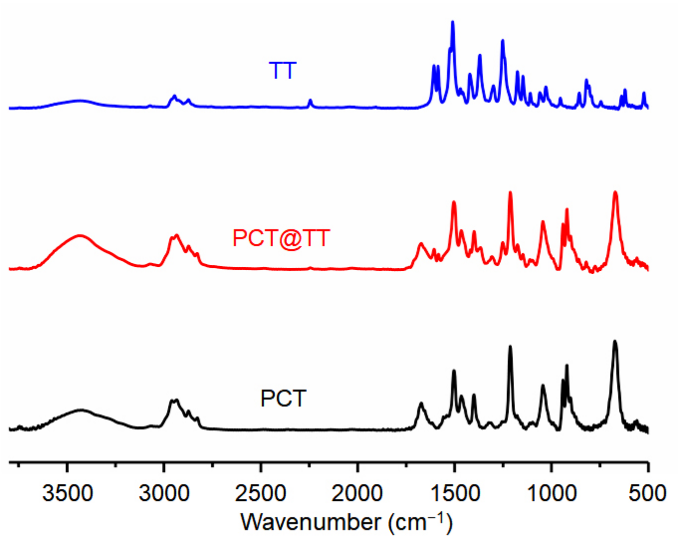

Supplementary Fig. 18 FT-IR spectra. TT, PCT, and PCT@TT precipitate.

Supplementary Table 2. Elemental analysis data of PCT@TT precipitates.

**$[PCT]_3[TT]_2$**	**C**	**N**	**H**
**Found (%)**	53.89	3.72	6.28
**Calcd. (%)**	53.96	3.84	6.49

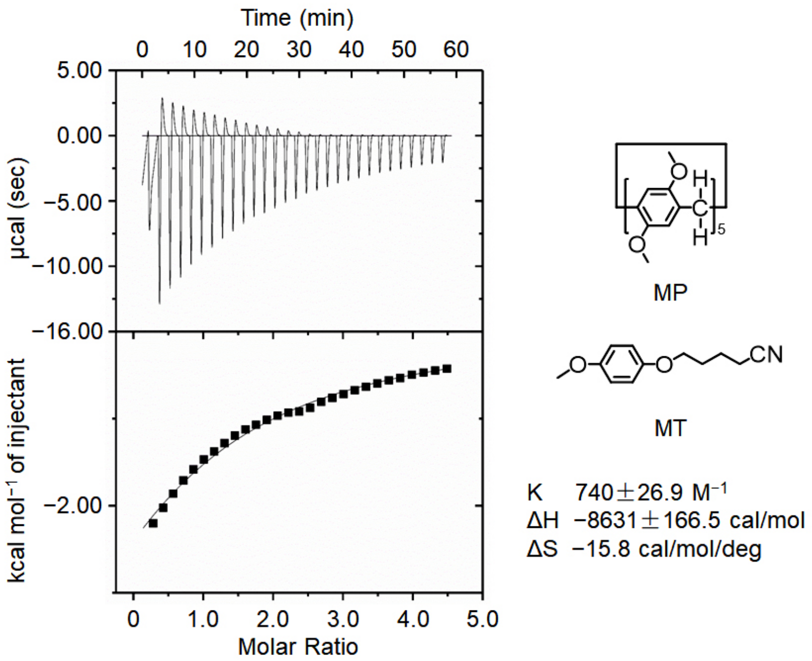

Supplementary Fig. 19 Biding model of MP and MT. ITC titration curve and fitted data of MP^5 to MT in chloroform at 25°C, which gives a 1:1 association constant K=740±26.9 M^{-1}. "±" values represent standard deviations.

Structural characterization of the supramolecular frameworks

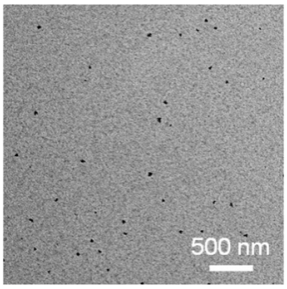

Supplementary Fig. 20 TEM image. PCT alone in chloroform (1 mg/mL).

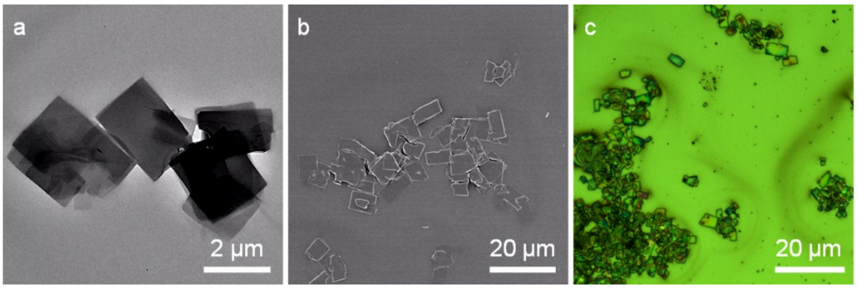

Supplementary Fig. 21 Morphologies of PCT@TT SFs. (a) TEM, **(b)** SEM, **(c)** reflection mode of polarizing microscope images in $CHCl_3$.

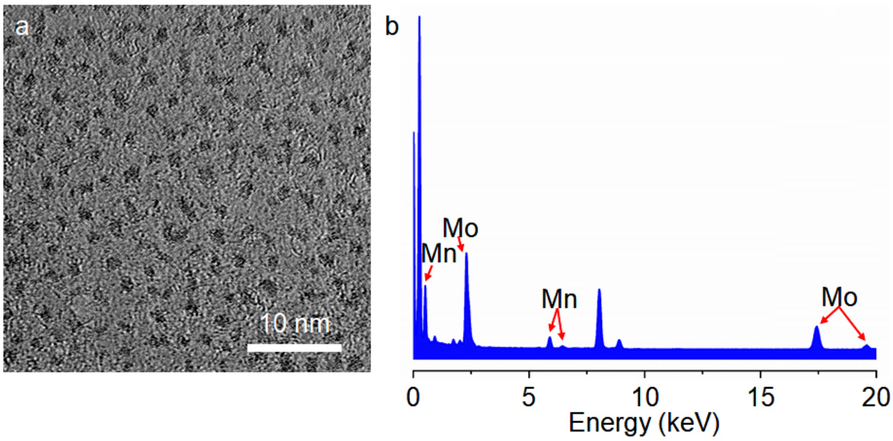

Supplementary Fig. 22 Characterization of PCT@TT SFs. (a) HR-TEM images of PCT@TT SFs, **(b)** Energy dispersive X-ray spectrum (EDX) focusing on PCT@TT SF assemblies.

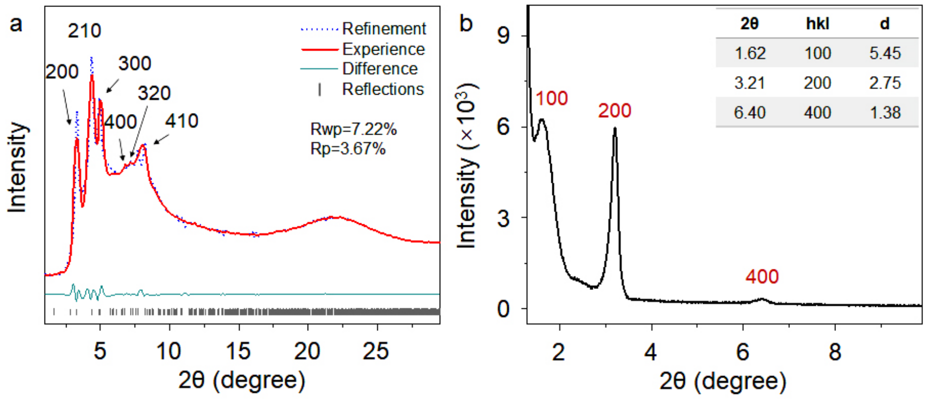

Supplementary Fig. 23 Powder XRD pattern. PCT@TT SFs. **(a)** Single crystal X-ray diffraction (SCXRD) pattern of PCT@TT SF in powder mode. Peaks are assigned in Supplementary Table 3. **(b)** Grazing incidence X-ray diffraction (GIXRD) of PCT@TT SF membrane.

Supplementary Table 3. Lists of the peak assignments found in the SCXRD spectrum of PCT@TT SFs in powder mode, shown in Supplementary Fig. 20a.

	2θ/°	θ/°	$\sin^2\theta \times 10^4$	h^2+hk+k^2	hkl	d/nm	FWHM[b]
1	3.20	1.60	7.80	4	200	2.76	0.0095
2	4.30	2.15	14.07	7	210	2.05	0.0118
3	5.15	2.57	20.11	9	300	1.72	0.0140
4	6.70	3.35	34.15	16	400	1.32	-
5	7.05	3.53	37.91	18	320	1.25	-
6	7.90	3.95	47.45	24	410	1.12	-
7	21.85	10.93	-	-	$d_{\pi\text{-}\pi}$[a]	0.41	-

[a] π-π distance of pillar[5]arene in adjacent layers. The full-width-at-half-maximum (FWHM) values can only be taken from the first three diffraction peaks. [b] radians.

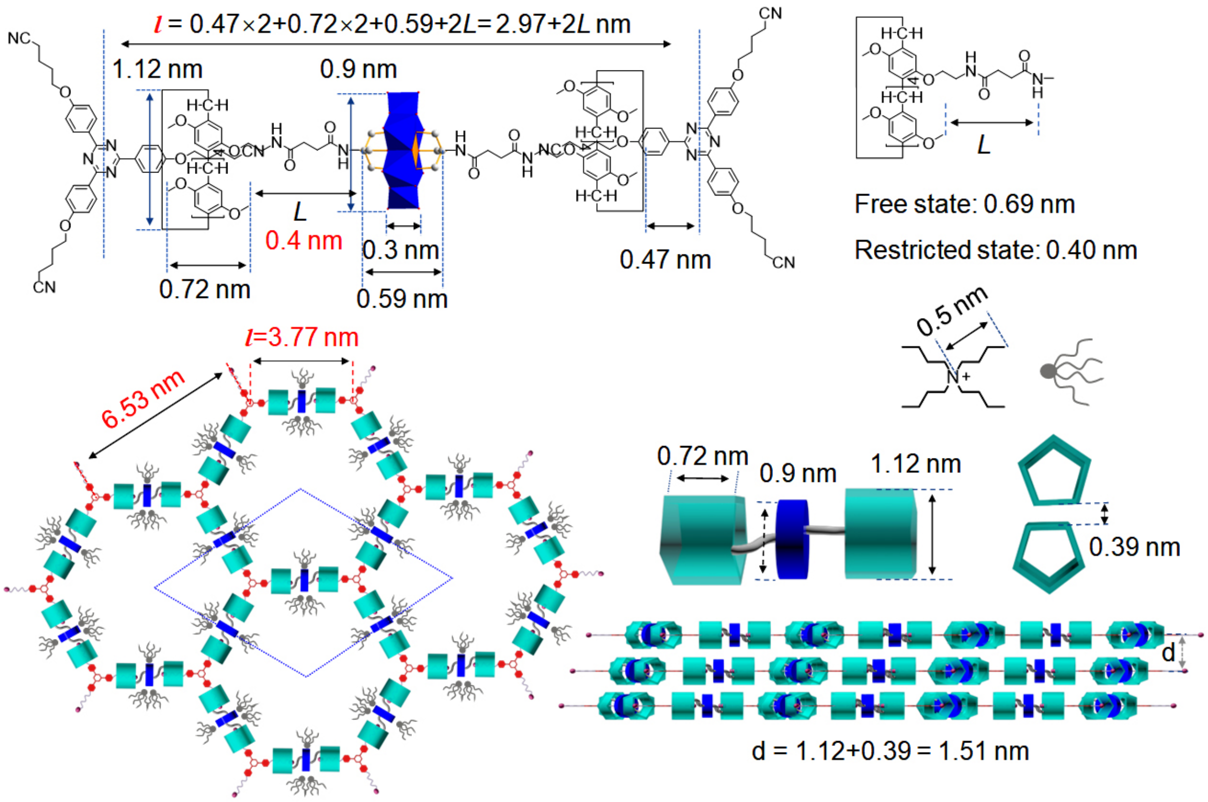

Supplementary Fig. 24 Structure matching of PCT@TT SFs. TBA cations are located inner the pores to reduce the exposure of the hydrophilic POMs in the hydrophobic solvents. Here, the length of flexible linker part *L* at a restricted state is 0.40 nm based on the simulation from Chem3D, while the other parts of the host-guesting binding geometry in length are extracted from crystal structure, giving an *l* value 3.77 nm. Following a hexagonal model, the calculated dimension is 6.53 nm. After considering the diameter of POM (0.9 nm), electrostatic interaction distance between POM and TBA (0.3 nm) and the size of TBA (0.5 nm), the pore size can be calculated to be 6.53-0.9-0.5×2-0.3×2 nm = 4.03 nm in diameter. The thickness of the single-layer SF is estimated to be 1.51 nm.

Solvent exfoliation of the supramolecular frameworks

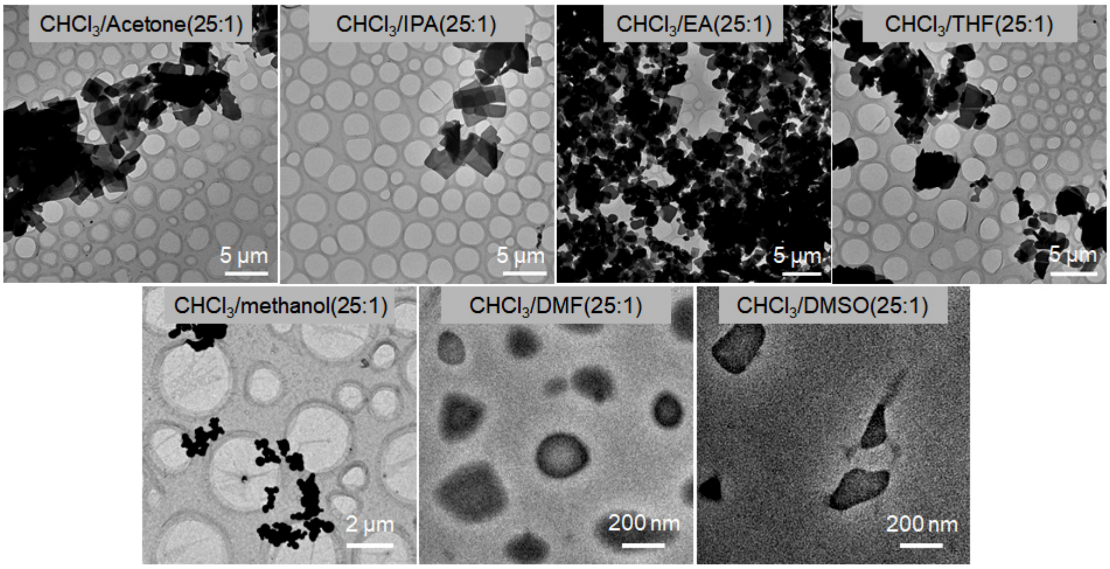

Supplementary Fig. 25 Morphologies of PCT@TT SFs. TEM images of PCT@TT SFs dispersed in different mixed solvents, IPA: isopropyl alcohol, EA: ethyl acetate, THF: tetrahydrofuran, DMF: dimethyl formamide, DMSO: dimethyl sulfoxide.

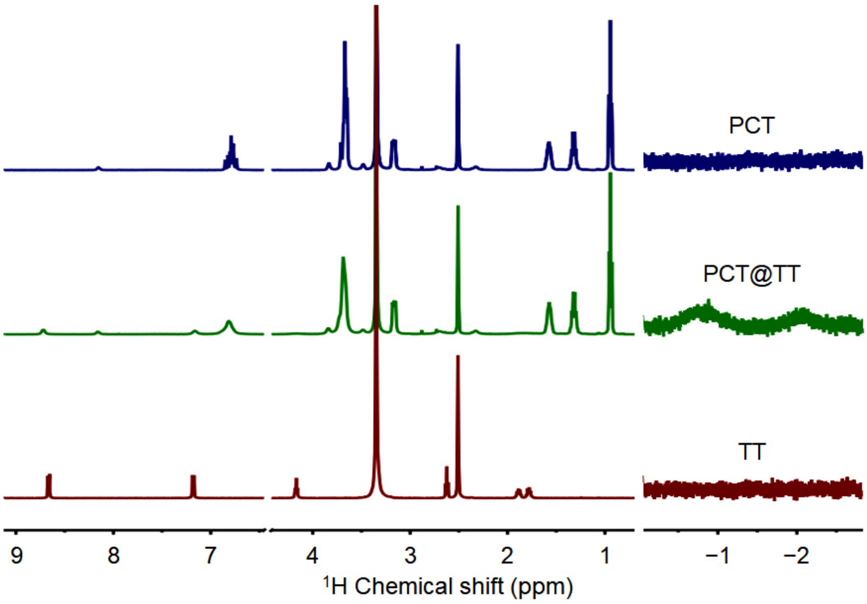

Supplementary Fig. 26 ^{1}H NMR spectra. TT, PCT@TT and PCT in DMSO-d_6/D_2O= 10:1 (500 MHz, 25°C).

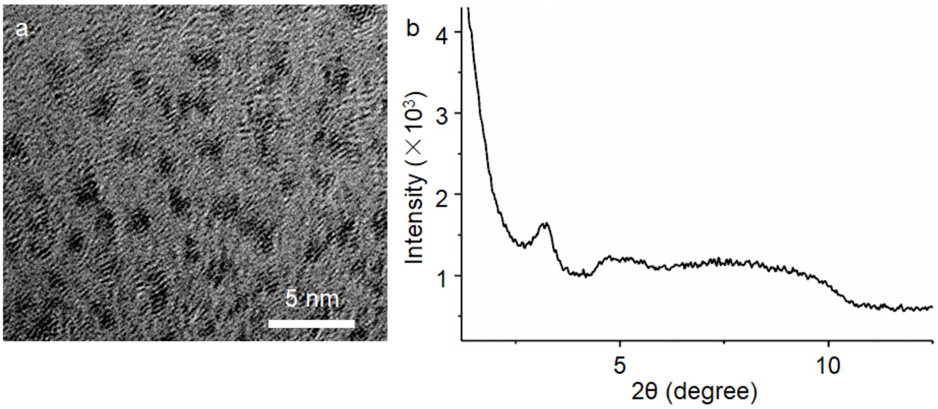

Supplementary Fig. 27 Characterization of exfoliated PCT@TT SFs. (a) HR-TEM image and **(b)** XRD pattern of exfoliated PCT@TT SFs dispersed in DMSO/H_2O (1:4, v/v).

Structure characterization of the supramolecular framework membrane

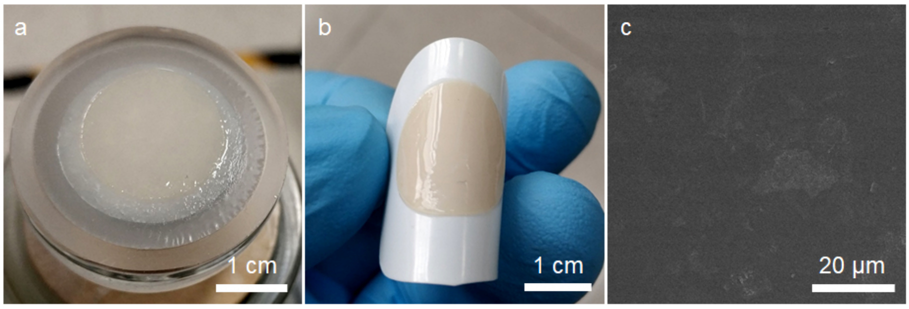

Supplementary Fig. 28 Flexibility of the membrane. Digital pictures **(a, b)** and SEM image **(c)** of the SF membrane.

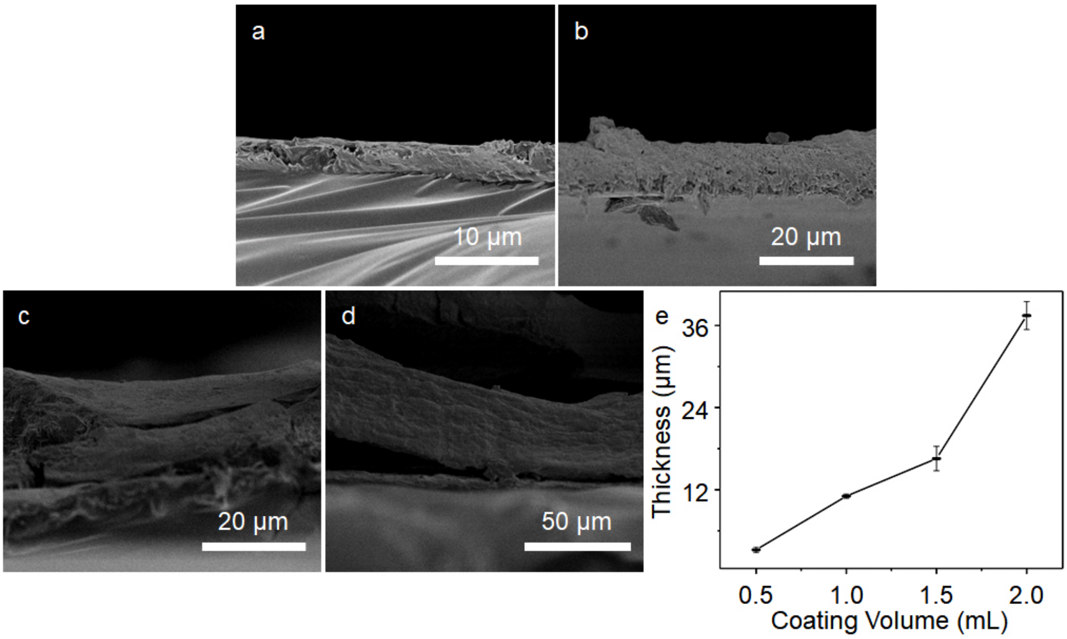

Supplementary Fig. 29 SEM images and thickness of membranes. Cross-sectional SEM images of membranes with different coating volumes: **(a)** 0.5 mL, **(b)** 1.0 mL, **(c)** 1.5 mL, **(d)** 2.0 mL of SFs solution. **(e)** Relationship of the thickness of membranes verse coating volume.

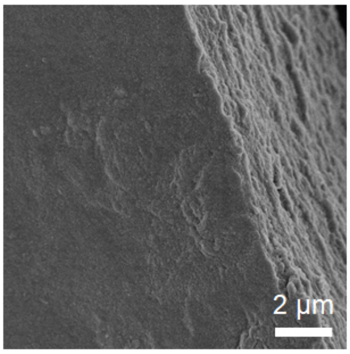

Supplementary Fig. 30 SEM images. Lateral view SEM image of the laminated membrane.

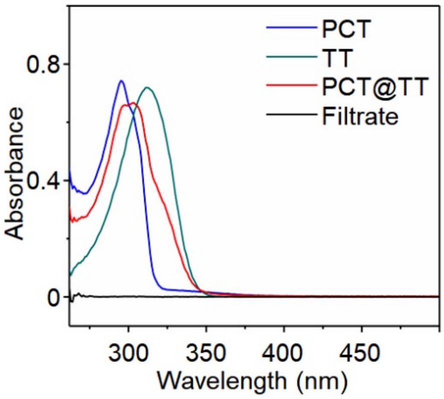

Supplementary Fig. 31 UV-vis spectra. The filtrate and components of PCT@TT SFs membrane.

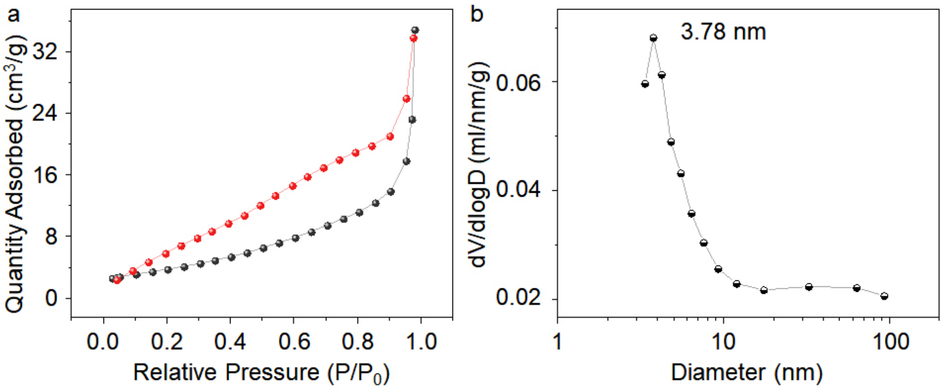

Supplementary Fig. 32 Evaluation of the pore. (a) N_2 adsorption/desorption isotherms of PCT@TT SF powders. **(b)** Pore size distribution calculated by density functional theory method from N_2 sorption isotherms for PCT@TT SF powders at 77K. It shows the most probable pore size distribution is 3.78 nm, matched well with the value calculated from the proposed hexagonal model.

Membrane separation of Au nanoparticles

Mercaptopropionic acid (MPA) stabilized gold nanoparticles were prepared according to a modified literature procedure.[6]

Au-MPA-1 (2.90±0.50 nm): $HAuCl_4 \cdot 3H_2O$ (3.0 mL, 14.5 mM) was added to 70 mL of refluxing doubly distilled water. Then, a mixture of MPA-Na (0.18 mL, 0.25 M) and trisodium citrate (3.0 mL, 0.17 M) was added rapidly with stirring. Then solution was refluxed for another 5 h. After the solution cooled down to the room temperature, the solution was filtrated with 220 nm filter membrane. HCl (10 mL, 2 M) was added to the filtrate and the particles were precipitated. The precipitate was collected by centrifugation and washed with HCl for 3 times. Then it was re-dispersed in 70 mL water with NaOH (0.2 mL, 1 M). Putting them in fridge (4°C) before use.

Au-MPA-2 (6.07±1.35 nm): $HAuCl_4 \cdot 3H_2O$ (3.0 mL, 14.5 mM) was added to 70 mL of refluxing doubly distilled water. Then, a mixture of MPA-Na (0.36 mL, 0.25 M) and trisodium citrate (3.0 mL, 0.17 M) was added rapidly with stirring. Then solution was refluxed for another 5 h. After the solution cooled down to the room temperature, the solution was filtrated with 220 nm filter membrane. HCl (10 mL, 2 M) was added to the filtrate and the particles were precipitated. The precipitate was collected by centrifugation and washed with HCl for 3 times. Then it was re-dispersed in 70 mL water with NaOH (0.2 mL, 1 M). Putting them in fridge (4°C) before use.

Au-GSH was prepared according to the literature procedure.[7] The freshly prepared Au-GSH has a negative Zeta potential, while the cationic Au-GSH can be obtained after the addition of the appropriate amount of hydrochloric acid (6M).

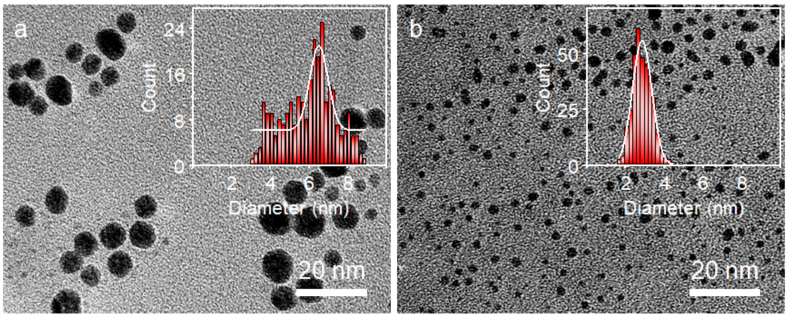

Supplementary Fig. 33 TEM images. (a) Au-MPA-1, **(b)** Au-MPA-2, where the insets are corresponding particle size histograms.

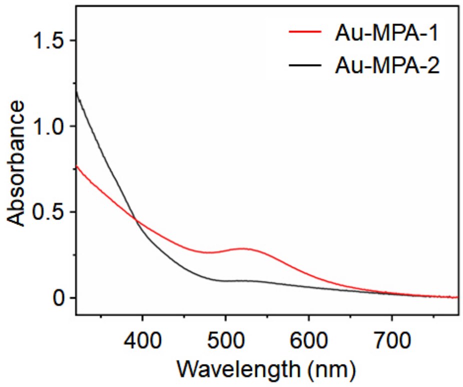

Supplementary Fig. 34 UV-vis spectra. Au-MPA-1 and Au-MPA-2.

Membrane separation of cationic and anionic dyes

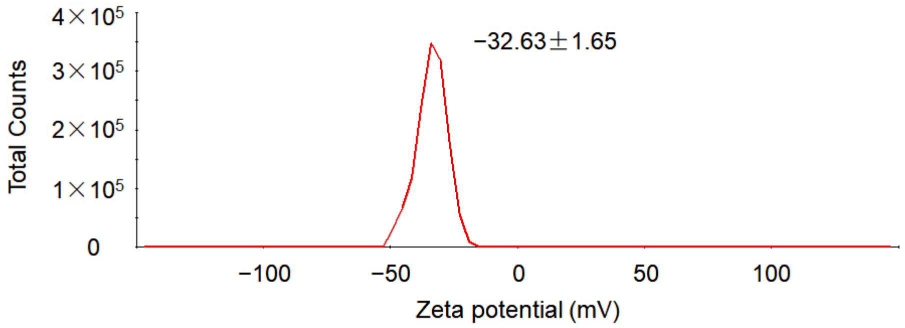

Supplementary Fig. 35 Zeta potential distribution. PCT@TT SF in DMSO/H_2O (2:8, v/v).

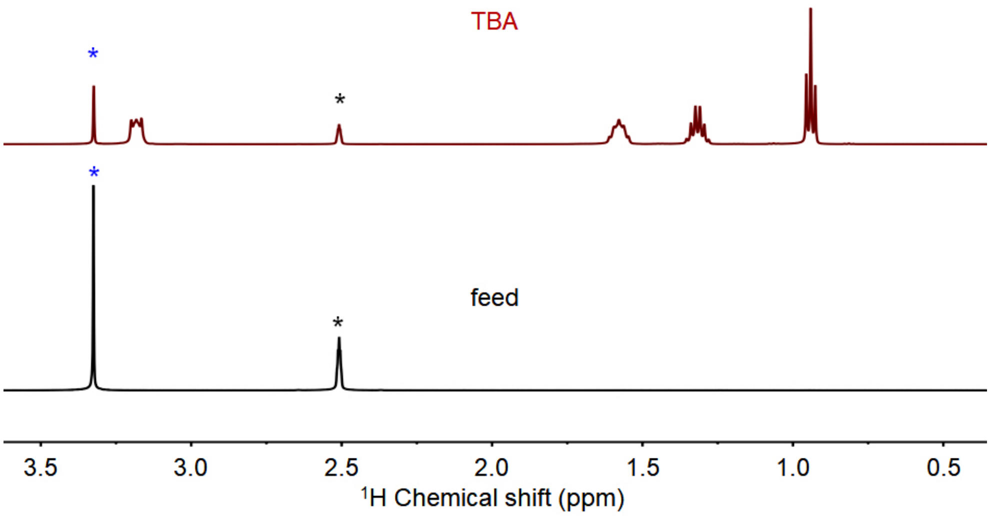

Supplementary Fig. 36 ^{1}H NMR spectra. TBA and filtrate of MB aqueous solution in DMSO-d_6 (500 MHz, 25°C). * is the peak of residual solvent DMSO, * is the peak of residual solvent water.

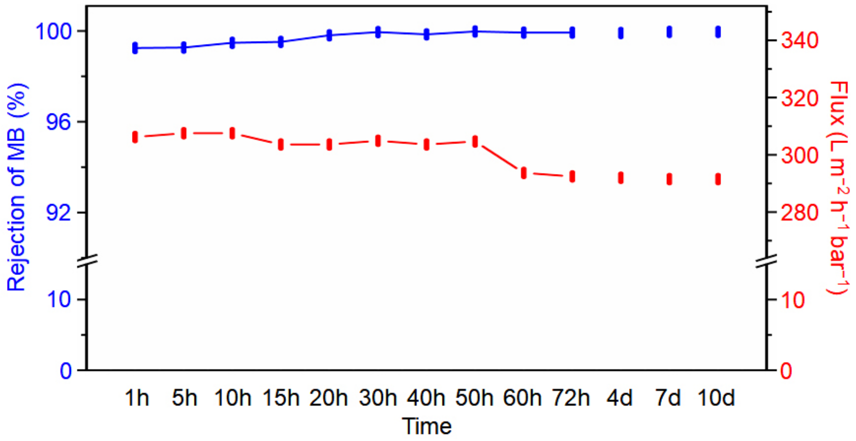

Supplementary Fig. 37 Separation performance of PCT@TT SFs for MB. Flux (red) and rejection (bule) efficiency versus separation time. The lined region means continuous separation of the dye solution and the dotted region is interrupted separation.

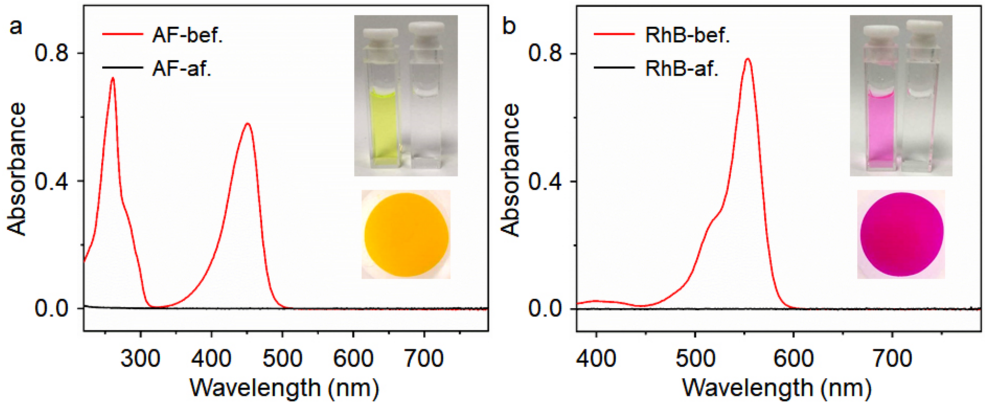

Supplementary Fig. 38 UV-vis spectra. Cationic dyes before and after separation: **(a)** AF (0.004 mg/mL), **(b)** RhB (0.08 mg/mL).

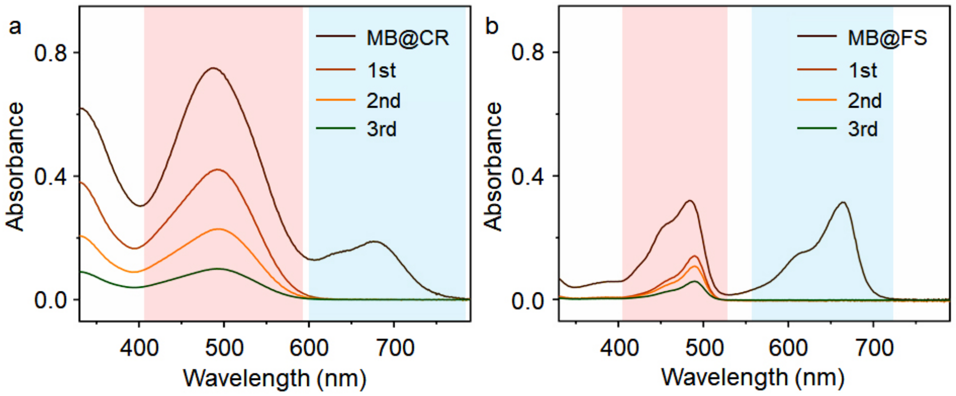

Supplementary Fig. 39 UV-vis spectra. Mixed dyes before and after separation: **(a)** MB@CR, **(b)** MB@FS.

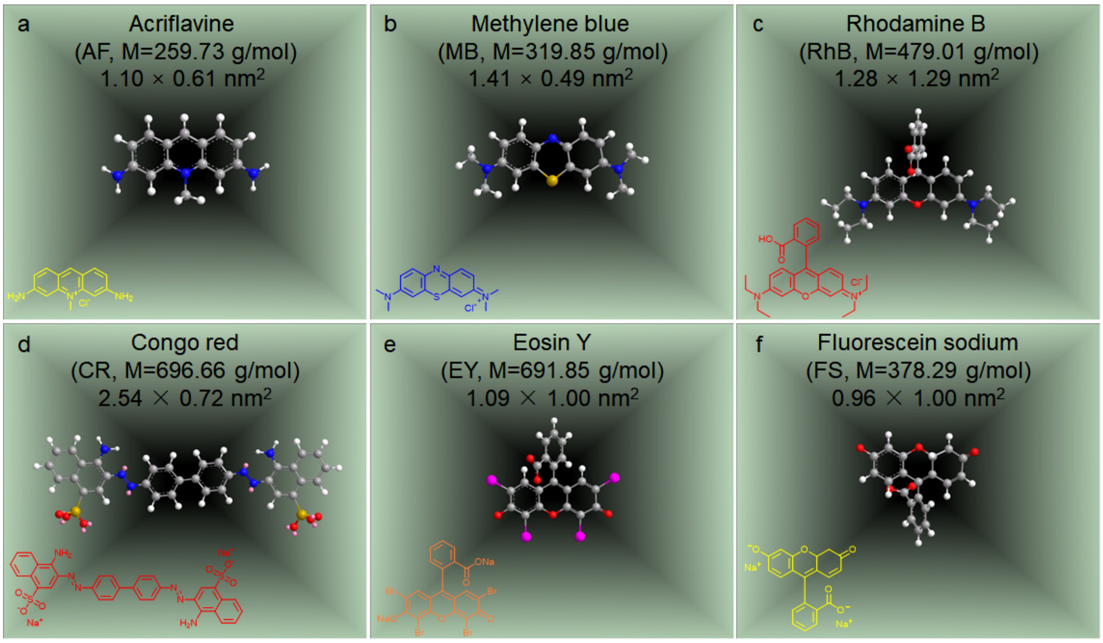

Supplementary Fig. 40 Chemical structure and geometric size in Chem3D. (a) AF, **(b)** MB, **(c)** RhB, **(d)** CR, **(e)** EY, **(f)** FS, counterions are omitted.

Protein separation by SF membrane

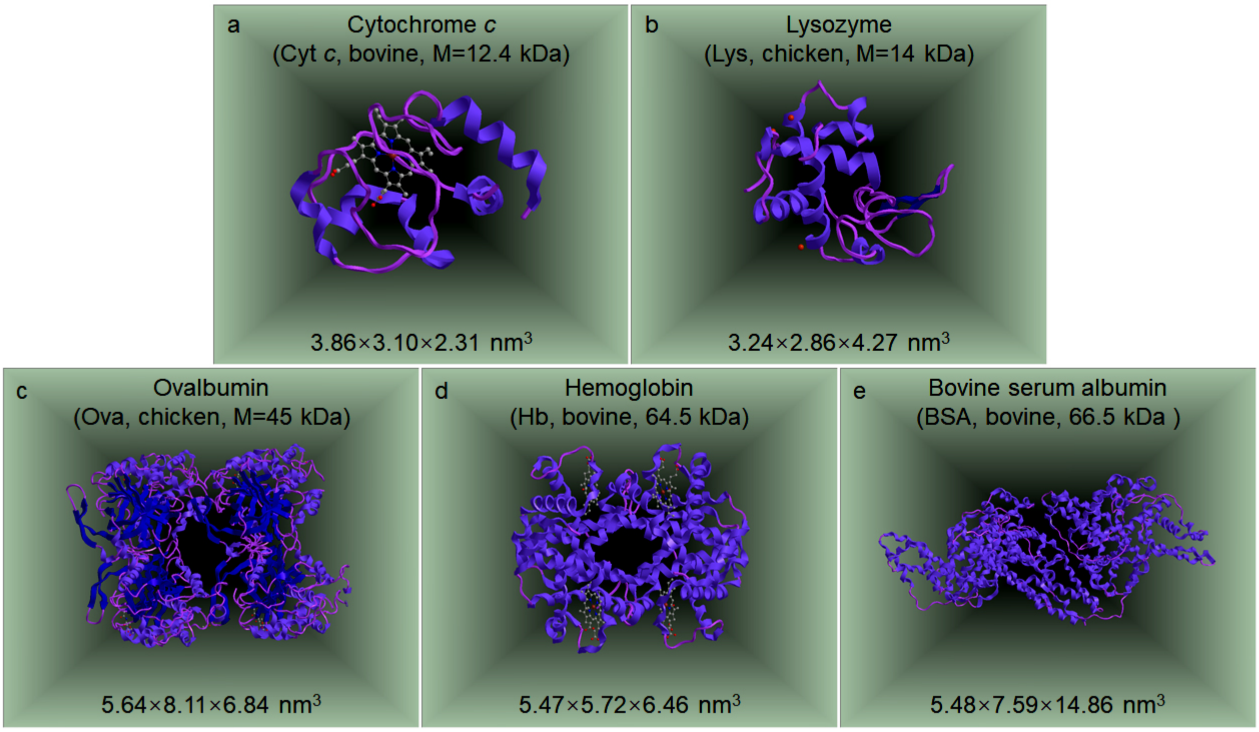

Supplementary Fig. 41 Crystal structure and geometric size of proteins. (a) Cyt *c*, bovine,[8] **(b)** Lys, chicken,[9] **(c)** Ova, chicken,[10] **(d)** Hb,[11] **(e)** BSA, bovine,[12] counterions are omitted.

Supplementary Table 4. Parameters of series substrates to be separated and separation performance.

Substrates	Size	Feed concentration	Solution environment	Surface potential	Permeation efficiency (%)
AF	1.10 × 0.61 nm^2 [a]	0.004 mg/mL	water	13.93±2.97	0.12±0.02
MB	1.41 × 0.49 nm^2 [a]	0.004 mg/mL	water	13.20±1.59	0.09±0.08
RhB	1.28 × 1.29 nm^2 [a]	0.08 mg/mL	water	1.92±0.19	0.11±0.07
CR	2.54 × 0.72 nm^2 [a]	0.4 mg/mL	water	−36.1±2.99	99.63±1.44 [b]
EY	1.09 × 1.00 nm^2 [a]	0.004 mg/mL	water	−22.93±4.41	99.05±1.05 [b]
FS	0.96 × 1.00 nm^2 [a]	0.006 mg/mL	water	−15.63±2.14	96.02±2.35 [b]
CR@MB	–	–	water	−45.7±1.08	98.92±1.27 [b]
	–				0.09±0.06 [b]
EY@MB	–	–	water	−10.47±1.26	98.93±1.40 [b]
	–				0.08±0.03 [b]
FS@MB	–	–	water	−10.51±0.71	95.91±3.29 [b]
	–				0.06±0.05 [b]
Au(+)	2.00±0.36 nm	–	acidic solution	38.07±4.63	0.30±0.20
Au(−)	2.05±0.32 nm	–	water	−17.53±1.42	93.01±0.13
Cyc *c*(+)	3.86×3.10×2.31 nm^3	0.1 mg/mL	water	6.07±0.33	0.93±0.40 [b]
Cyc *c*(−)	–	0.1 mg/mL	alkaline solution	−42.5±2.17	94.28±4.63 [b]
Lys(+)	3.24×2.86×4.27 nm^3	0.2 mg/mL	water	12.23±1.85	0.36±0.07 [b]
Lys(−)	–	0.2 mg/mL	alkaline solution	−37.33±1.29	93.89±5.06 [b]
Hb(+)	5.47×5.72×6.46 nm^3	0.1 mg/mL	water	7.90±1.08	0.18±0.09 [b]
Hb(−)	–	0.2 mg/mL	alkaline solution	−51.30±2.95	3.14±0.06 [b]
Ova	5.64×8.11×6.84 nm^3	0.6 mg/mL	water	−9.46±0.21	4.69±0.02 [b]
BSA	5.48×7.59×14.86 nm^3	0.8 mg/mL	water	−21.73±1.46	2.59±0.24 [b]
Hb@Lys	–	–	alkaline solution	−48.00±4.16	94.47±2.49 [b]
	–				5.44±0.25 [b]
Ova@Cyt *c*	–	–	alkaline solution	−48.50±2.21	94.54±1.98 [b]
	–				1.72±0.16 [b]
BSA@Cyt *c*	–	–	alkaline solution	−40.13±3.61	91.03±1.14 [b]
	–				9.24±0.30 [b]

[a] size calculated by Chem3D. [b] efficiency data collected after 3 times separation.

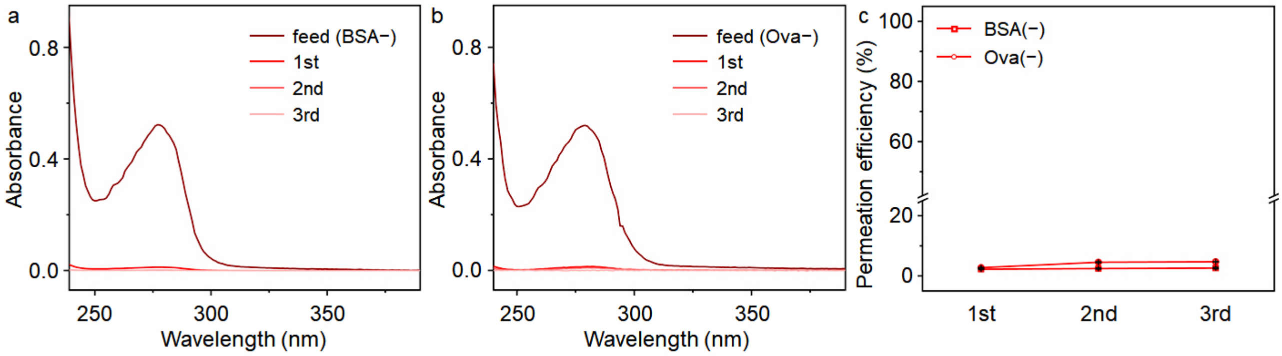

Supplementary Fig. 42 Separation of negative charged BSA (−) and Ova (−). UV-vis spectra of **(a)** BSA (−) in negative charge and **(b)** Ova (−) in negative charge before and after filtration, and **(c)** permeation efficiency of negative charged BSA and Ova during the separation process.

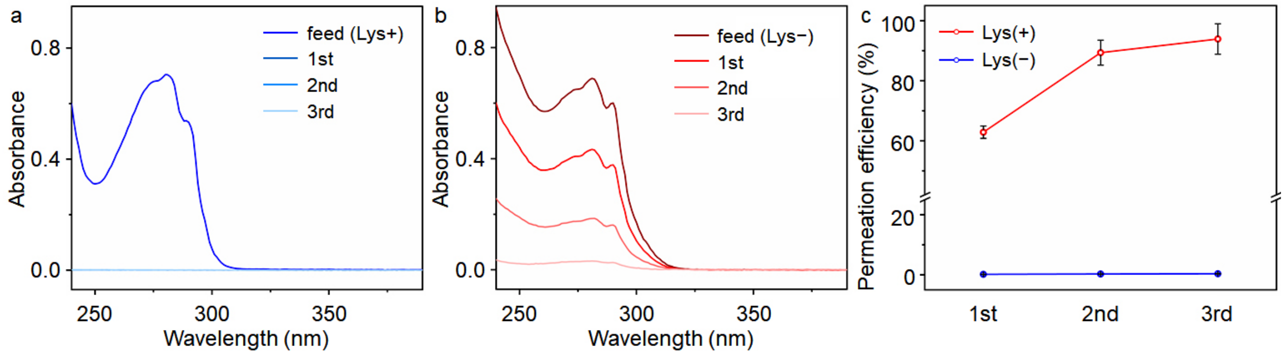

Supplementary Fig. 43 Separation of charged Lys. UV-vis spectra of **(a)** positive (+) and **(b)** negative charged (−) Lys before and after filtration, and **(c)** permeation efficiency of Lys during the separation process.

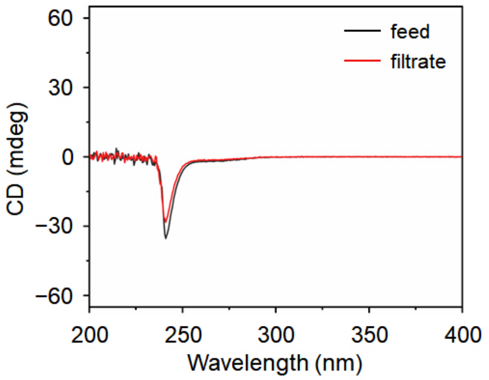

Supplementary Fig. 44 Structure evaluation of protein. CD spectra of BSA before and after passing through the membrane, in which the filtrate is collected from multiple separations and concentrated by freeze-drying and redissolved.

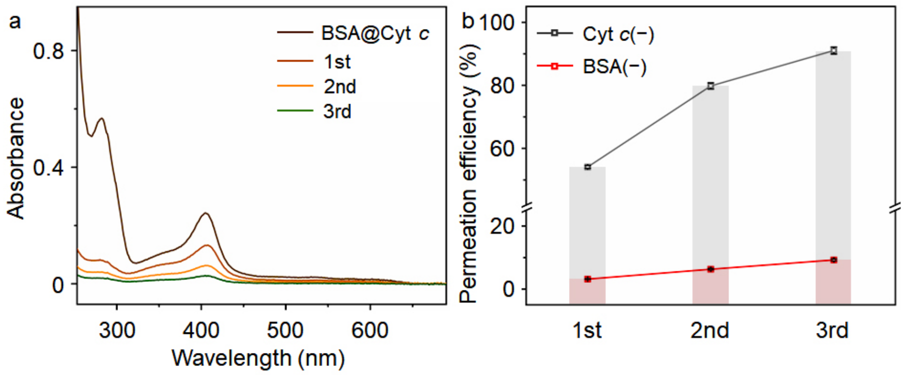

Supplementary Fig. 45 Separation of BSA@Cyt *c*. (a) UV-vis spectra and **(b)** permeation efficiency of negative charged BSA@Cyt *c* mixture solution during the separation process.

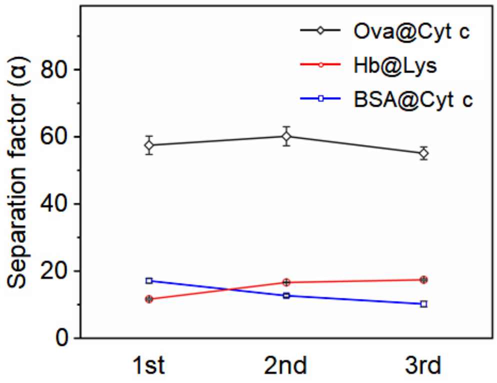

Supplementary Fig. 46 Evaluation of separation capability. Separation factor (α) plots of series protein mixtures versus the filtration cycles.

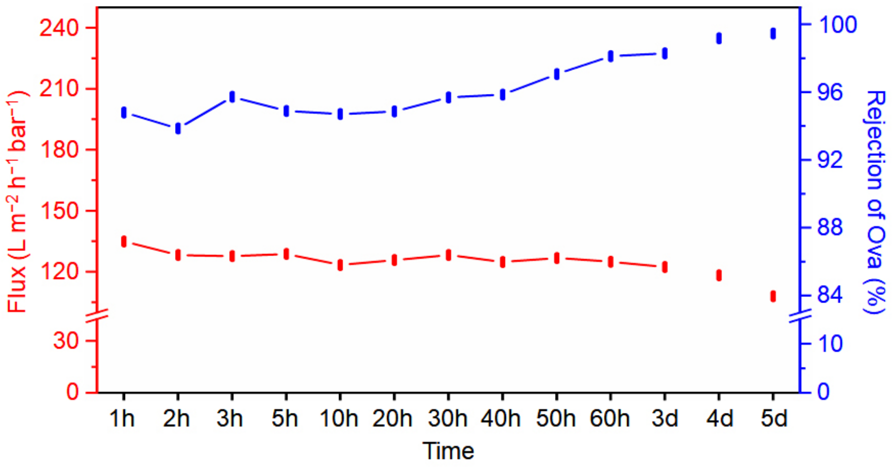

Supplementary Fig. 47 Anti-fouling performance of SF membrane for Ova (1×10^{-4} mg/L, room temperature). Flux and rejection efficiency plots versus separation time. The lined region means continuous separation of the proteins solution and the dotted region is interrupted separation.

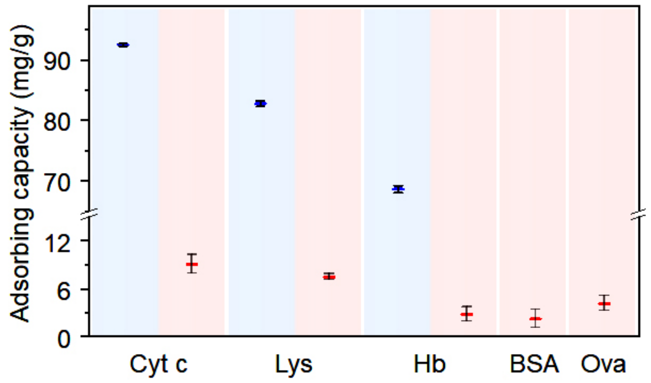

Supplementary Fig. 48 Static adsorbing capacity of SF assemblies for proteins. The bule and red domains mean the proteins exhibiting positive and negative surface potential, respectively.

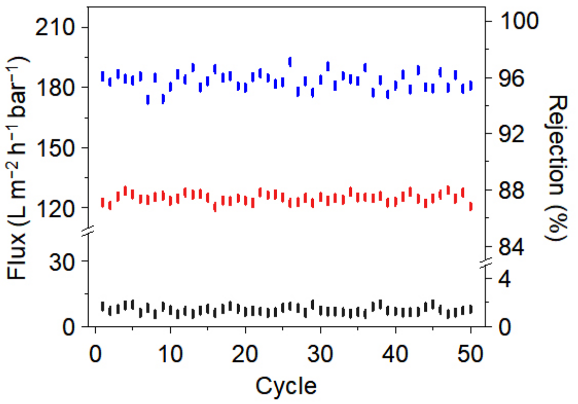

Supplementary Fig. 49 Reutilization performance of SF membrane for protein separation. Flux (red) and rejection efficiency values of Ova (in bule) and Cyt *c* (in gray) versus the reused filtration cycles.

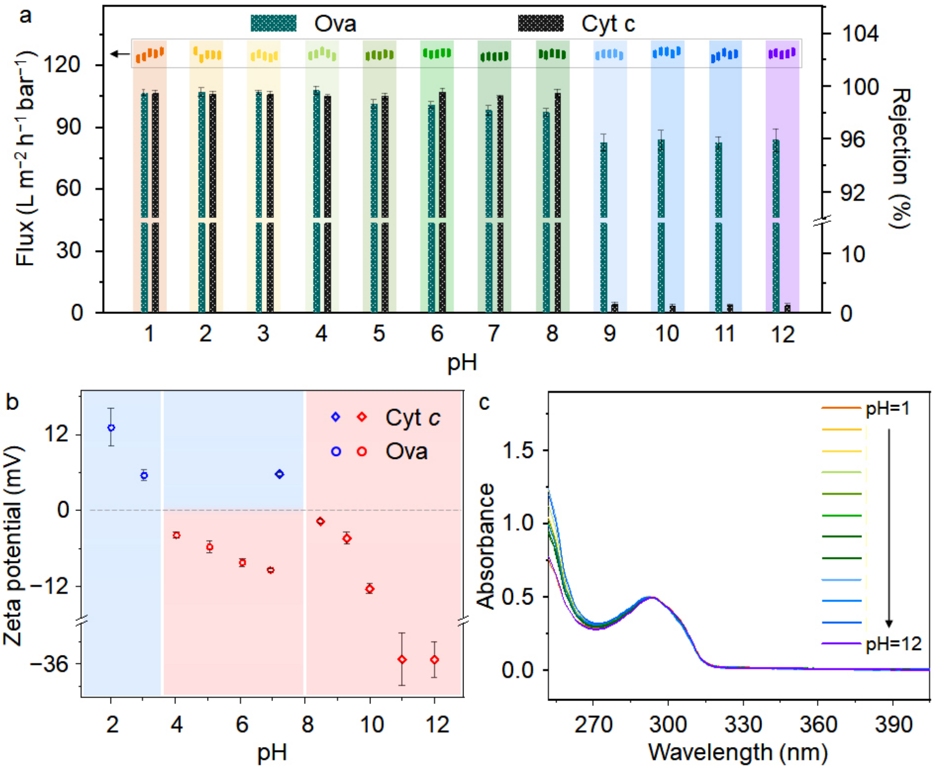

Supplementary Fig. 50 pH stability of SF membrane for protein separation. (a) Flux and rejection efficiency of protein solutions under different pH environment. **(b)** Zeta potential of protein Cyt c and Ova in different pH aqueous solution. **(c)** UV-vis spectra of PCT after treated with different pH aqueous solutions.

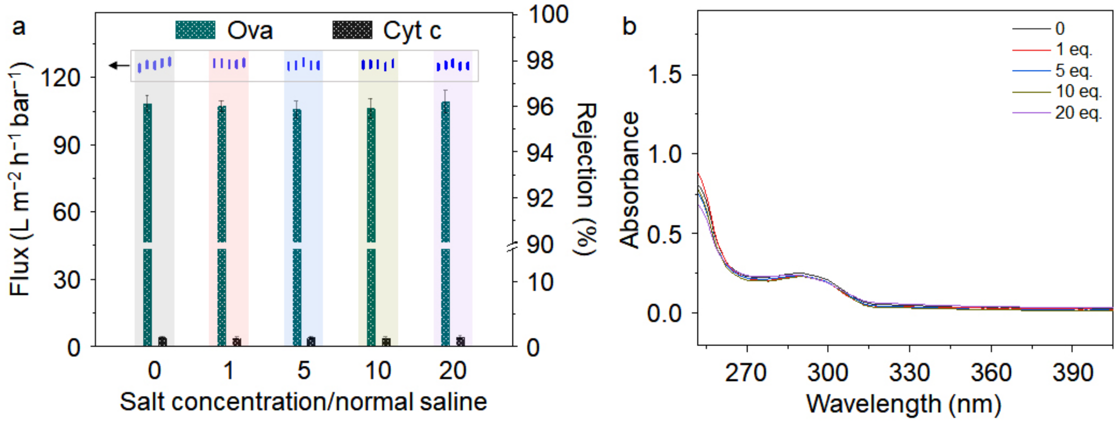

Supplementary Fig. 51 Salt stability of SF membrane for protein separation. (a) Flux and rejection efficiency under salt solution with different concentration. **(b)** UV-vis spectra of PCT after treated with different concentration of salt solutions.

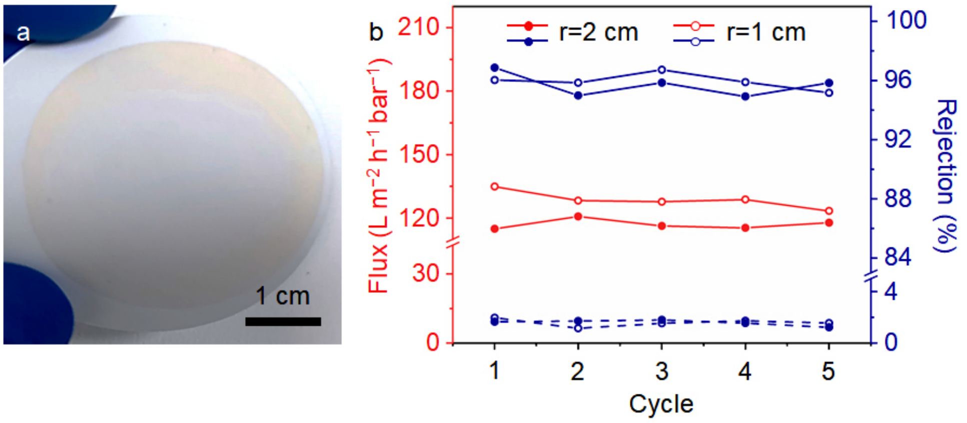

Supplementary Fig. 52 Separation performance of enlarged SF membrane. (a) Digital picture. **(b)** Flux (red line) and rejection efficiency (Ova, bule solid line; Cyt c, bule dash line) of versus separation cycles for different size membranes.

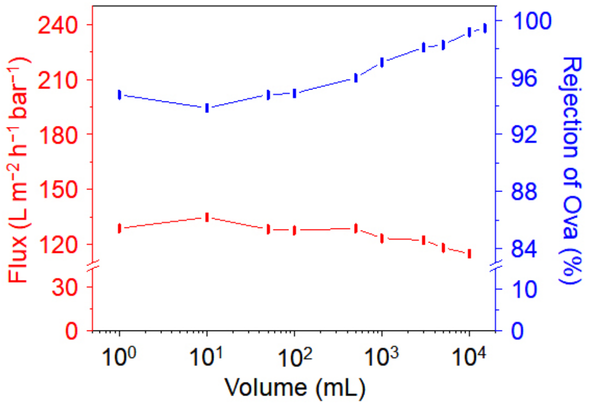

Supplementary Fig. 53 Separation performance of SF membrane for dose of substance to be separated. Flux and rejection efficiency of the membrane for separation Ova at concentration of 1×10^{-4} mg/L.

Supplementary Table 5. Summary and comparison of microporous framework membranes for size-dependent separation (in water).

Materials	Name	Pore size (nm)	Thickness	Cut-off (nm)	Rejection efficiency (%)	Flux (L m^{-2} h^{-1} bar^{-1})	pH	Ref.
SF	PCT@TT	3.9	3.6 μm	3.8	90.72	149.48±6.15	1–12	This work
			16.5 μm		99.80	36.39±1.19		
PPA	UTC 20 [a]	0.43	250 μm	0.41	95	15	3–10	13
PES	NF-PES-010 [a]	1	300 μm	N/A	90	5–10	0–14	
sPES	NTR 7450 [a]	0.79	200 μm	0.81	60	12	2–11	
PA	Desal-HL-51 [a]	0.48	250 μm	N/A	90	9	3–9	
PA	NF270 [a]	0.34	200 μm	N/A	99	11	3–9	
MA	mesoporous silica	9.4	50 nm	5	93.8	N/A	N/A	14
Protein	TMVm	4	43 nm	5.5	99.9	7460	6–10	15
SIF	ICOM@TCBP	1.92	50 μm	2.2	98.2	8957.0	N/A	16
$Pt_3(OTf)_3$	2D SP_{Pt3}	~3.8	0.6 μm	3.5	90	151±7	N/A	17
MOP	ZrT-1-NH_2/TMC	0.71	~50 nm	~0.76	99.7(RhB)	82.0	N/A	18
MOF	ML-UiO-66	0.628	103 nm	N/A	99.9	29.8	N/A	19
	Cu-TCPP SN	1.2	300 nm	N/A	99.8 (CR)	840.1	N/A	20
	ZIF-8/PSS	0.42	1.5 μm	N/A	98.6	26.5	N/A	21
POC	CC3-PAN	N/A	80 nm	N/A	99.73 (CR)	43.0	N/A	22
COF	TFP−PDA	1.59	120 nm	1.4	N/A	411	N/A	23
	Tp-Bpy	2.5	2.1 μm	N/A	97(AF)	211	N/A	24
	COF-LZU1	~1.8	400 nm	1.2	>90	76	N/A	25
GO	ZnO/rGO	~4	5 μm	N/A	98.1 (MB)	225	2−8	26
	HLGO	N/A	8 nm	N/A	99.9 (MB)	~5	N/A	27
BCP	PAP-PES	0.5	N/A	N/A	99.9 (MB)	64.8	N/A	28
CMP	CNT-EP-PC15	1.8	416 nm	N/A	95	28	N/A	29
CD	Polyamide-CD	0.61	~6 nm	0.61	>99 (BB)	~25	N/A	30
CB	DETA-1.2-CB_6-0.06	0.4 nm	80−115 nm	N/A	70.9 (IPA)	4.2	N/A	31

[a] Commercial polymer membrane. SF, supramolecular framework; PAA, polypiperazineamide; PES, polyethersulfon; sPES, sulfonated polyethersulfon; PA, polyamide; MA, mesoporous silica; SIF, supramolecular ionic framework; MOP metal-organic polyhedron; MOF, metal organic framework; POC, porous organic cages; COF, covalent organic framework; GO, graphene oxide; BCP, block copolymers; CMP, conjugated microporous polymers; CD, Cyclodextrin; CB, Cucurbituril. RhB, rhodamine B; CR, Congo red; AF, acriflavine; MB, methylene blue; BB, Brilliant blue R; IPA, isopropanol.

Supplementary Table 6. Summary and comparison of microporous framework membranes for charge-dependent separation.

Materials	Name	Pore size (nm)	Thickness	Charge	Rejection efficiency (%)	Flux (L m^{-2} h^{-1} bar^{-1})	Ref.
SF	PCT@TT	3.9	16.5 μm	−	MB (99.91±0.08)	294.99±12.47	This work
					AF (99.88±0.02)	297.09±16.00	
					RhB (99.89±0.07)	302.67±7.30	
MOF	MIL-100(Cr) PE MMM-86%	N/A	N/A	−	CV (99.0)	112.5	32
					RhB (99.2)	108	
					MB (99.2)	120	
	NH2-UiO-66 PE MMM-86%	N/A	N/A	+	FA (99.1)	111.4	
					OG (99.0)	115.9	
COF	TpPa-SO_3Na	1.4	2.8 μm	−	MB (99.8)	~310	33
GO	ZnO/rGO	~4	5 μm	−	MB (98.1)	225	26
MPCM	MPCM	0.38	17−20 nm	+	OG (96)	35	34

SF, supramolecular framework; MOF, metal organic framework; COF, covalent organic framework; GO, graphene oxide; MPCM, molecularly porous cross-linked membranes. MB, methylene blue; AF, acriflavine; RhB, rhodamine B; CV, crystal violet; OG, crystal violet.

Supplementary Table 7. Summary and comparison of microporous framework membranes for protein separation.

Materials	Name	Pore size (nm)	Thickness	Charge	Rejection efficiency (%)	Separation factor	Flux (L m^{-2} h^{-1} bar^{-1})	pH	Ref.
SF	PCT@TT	3.9	16.5 μm	−	97.41±0.76 (BSA)	10.2 (BSA/Cyt *c*)	125.74±2.07	1–12	This work
					95.31±0.98 (Ova)	17.4 (Hb/Lys)	124.67±2.21		
					99.82±0.91 (Hb)	55.1 (Ova/Cyt *c*)	128.24±0.43		
MOF	MIL-100(Cr) PE MMM	N/A	N/A	−	N/A	94 (BSA/Hb)	N/A	N/A	32
Protein	PLT	~3.1	50 nm	+	98.4 (BSA)	N/A	226 ± 17	1−12	35
BCP	PS-b-P4VP	N/A	400 nm	+	N/A	87 (BSA/IgG)	N/A	4−11	36
						10 (BSA/Hb)			
	C-38	18.5	38 nm	+	92.2 ± 1.1 (Lys)	5.5 (Lys/LG)	210.3 ± 11.3	4–7.5	37
					57.1 ± 8.4 (LG)				
SAMs	PCTE/Au/ SAMs	11	N/A	N/A	N/A	67 (BSA/Hb)	N/A	N/A	38
PVDF	FP100 [a]	N/A	N/A	N/A	N/A	56.6 (BSA/IgG)	~13.5	4.7–8.5	39
RC	YM 100 [a]	14	N/A	−	97.3 (LG)	N/A	N/A	4.3–9	40

[a] Commercial polymer membrane. SF, supramolecular framework; MOF, metal organic framework; BCP, block copolymers; SAMs, self-assembled monolayers. PVDF, polyvinylidene fluoride; RC, regenerated cellulose; BSA, bovine serum albumin; Ova, ovalbumin; Hb, hemoglobin; Cyt *c*, cytochrome *c*; IgG, immunoglobulin-γ; Lys, lysozyme; LG, β-lactoglobulin.

Additional evaluations for SF membrane analysis

To block the possible channels at the grain boundary, a regularly used method was adopted for a further evaluation. By mixing the SF assembly with a polymer (polycaprolactone, PCL) up to a weight percent of 50%, the separation of the prepared composite membrane was repeated. The size-dependent separation of model Au NPs presents a cut-off value of ca. 4.0 nm almost identical to the isolated SF membrane. By contrast, both pure PCL polymer and its composite membrane with graphene at content of 50 wt% displayed no permeation for the same feed solution under the identical filtration pressure, demonstrate the effectiveness of PCL in covering granular gaps. The result clearly confirmed that the framework pores from SF assemblies, instead of the intergranular pores, play a main role in the size-dependent separation.

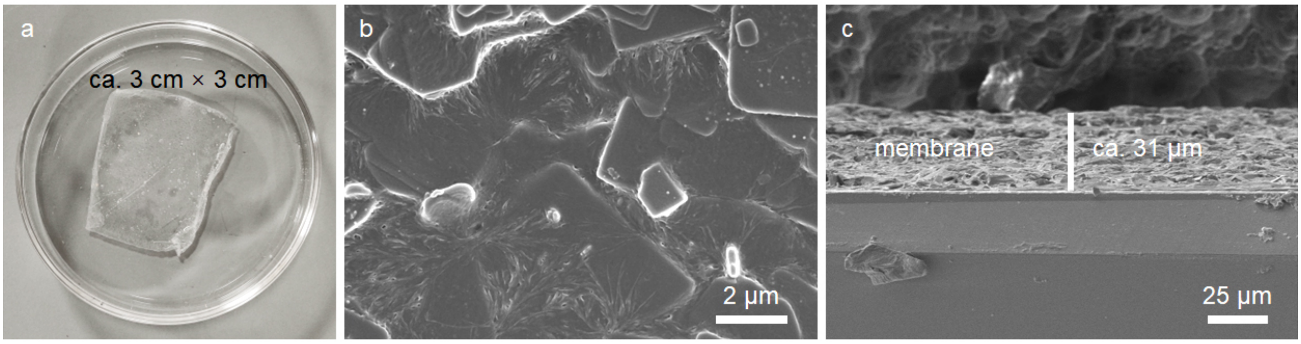

Supplementary Fig. 54 Morphology of SF-PCL membrane for separation of AuNPs. (**a**) Digital picture of SF-PCL membrane prepared with a mass ratio of 1:1. (**b**) SEM image of the membrane in top view. (**c**) SEM image of the membrane in side view.

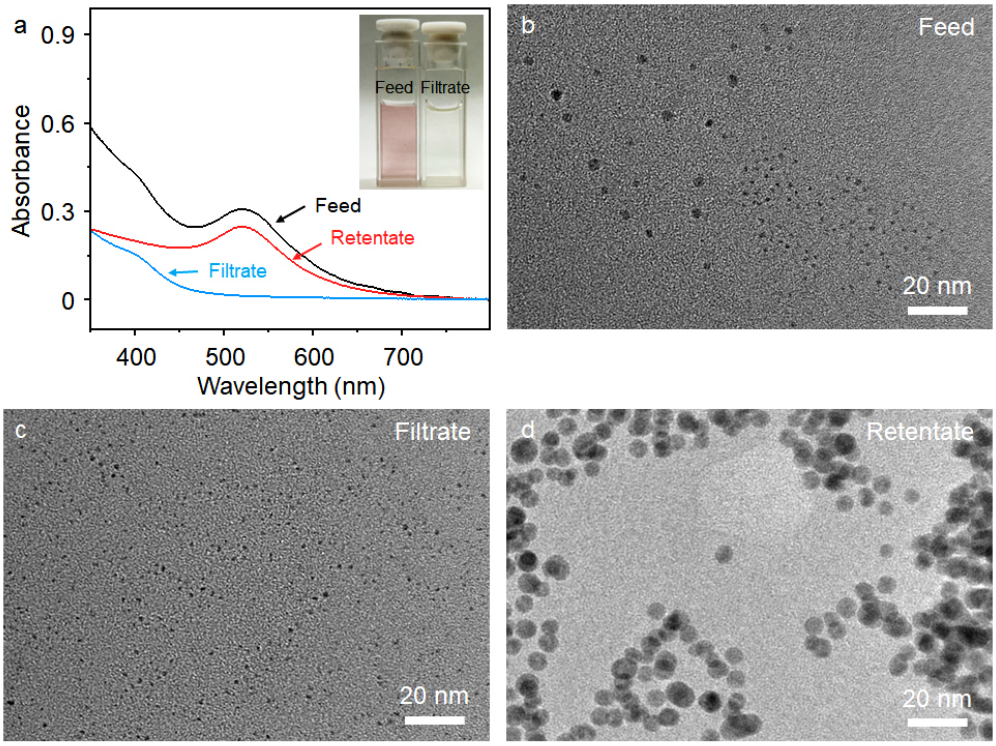

Supplementary Fig. 55 Size characterization of AuNPs separated by SF-PCL membrane. (**a**) UV-Vis spectra of Au NPs in aqueous solutions before and after filtration. (**b**) TEM image of feed. (**c**) TEM image of filtrate. (**d**) TEM image of retentate.

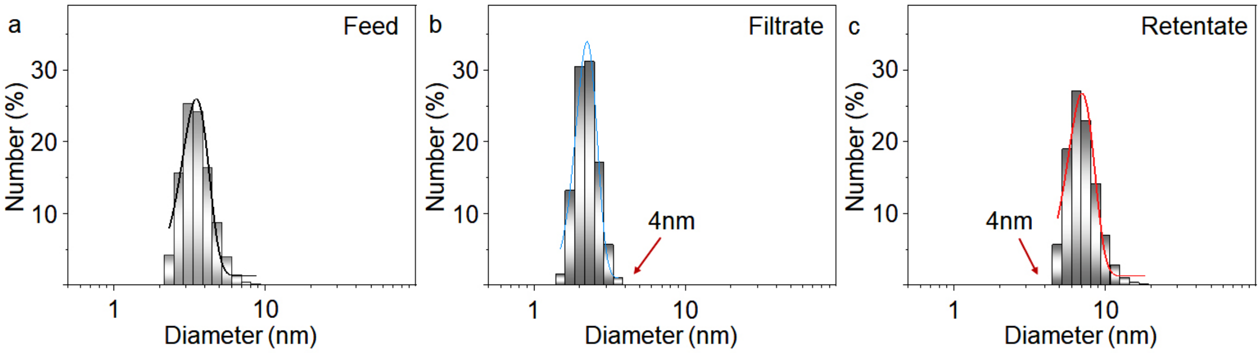

Supplementary Fig. 56 Statistical analysis of AuNPs for separation of SF-PCL membrane. DLS plot of (**a**) feed**,** (**b**) filtrate, and (**c**) retentate solution.

Supplementary references

1 Gao, L., Dong, S., Zheng, B. & Huang, F. Synthesis of a pillar[5]arene-based heteroditopic host and its complexation with n-octyltriethylammonium salts. *Eur. J. Org. Chem.* **2013**, 1209−1213 (2013).

2 Yvon, C. *et al.* Polyoxometalate clusters integrated into peptide chains and as inorganic amino acids: Solution- and solid-phase approaches. *Angew. Chem. Int. Ed.* **53**, 3336−3341 (2014).

3 Song, Y. F., Long, D. L. & Cronin, L. Noncovalently connected frameworks with nanoscale channels assembled from a tethered polyoxometalate-pyrene hybrid. *Angew. Chem. Int. Ed.* **46**, 3900−3904 (2007).

4 Liu, D. J. K., Zhang, G. H., Gao, B., Li, B. & Wu, L. X. From achiral to helical bilayer self-assemblies of a 1,3,5-triazine-2,4,6-triphenol-grafted polyanionic cluster: Countercation and solvent modulation. *Dalton trans.* **48**, 11623−11627 (2019).

5 Ogoshi, T., Kanai, S., Fujinami, S., Yamagishi, T. A. & Nakamoto, Y. Para-bridged symmetrical pillar[5]arenes: Their lewis acid catalyzed synthesis and host-guest property. *J. Am. Chem. Soc.* **130**, 5022−5023 (2008).

6 Yonezawa, T. & Kunitake, T. Practical preparation of anionic mercapto ligand-stabilized gold nanoparticles and their immobilization. *Colloids and Surfaces A: Physicochemical and Engineering Aspects* **149**, 193−199 (1999).

7 Fu, D.-Y. *et al.* Strong red-emitting gold nanoclusters protected by glutathione S-transferase. *Nanoscale* **10**, 23141−23148 (2018).

8 Mirkin, N., Jaconcic, J., Stojanoff, V. & Moreno, A. High resolution x-ray crystallographic structure of bovine heart cytochrome *c* and its application to the design of an electron transfer biosensor. *Proteins* **70**, 83−92 (2008).

9 Lim, K., Nadarajah, A., Forsythe, E. L. & Pusey, M. L. Locations of bromide ions in tetragonal lysozyme crystals. *Acta Crystallogr D Biol Crystallogr* **54**, 899−904 (1998).

10 Stein, P. E., Leslie, A. G. W., Finch, J. T. & Carrell, R. W. Crystal structure of uncleaved ovalbumin at 1·95 Å resolution. *J. Mol. Biol.* **221**, 941−959 (1991).

11 Aranda, R. t. *et al.* Structural analysis of fish versus mammalian hemoglobins: Effect of the heme pocket environment on autooxidation and hemin loss. *Proteins* **75**, 217−230 (2009).

12 Bujacz, A. Structures of bovine, equine and leporine serum albumin. *Acta Crystallogr D Biol Crystallogr* **68**, 1278−1289 (2012).

13 Braeken, L. *et al.* Transport mechanisms of dissolved organic compounds in aqueous solution during nanofiltration. *J. Membr. Sci.* **279**, 311−319 (2006).

14 Liu, Y. *et al.* Mesoporous silica thin membranes with large vertical mesochannels for nanosize-based separation. *Adv. Mater.* **29**, 1702274 (2017).

15 Zhang, S. *et al.* Ultralarge single-layer porous protein nanosheet for precise nanosize separation. *Nano Lett.* **18**, 6563−6569 (2018).

16 Zhou, Y., Zhang, G., Li, B. & Wu, L. Two-dimensional supramolecular ionic frameworks for precise membrane separation of small nanoparticles. *ACS Appl. Mater. Interfaces.* **12**, 30761−30769 (2020).

17 Chen, Z. & Yam, V. W. Precise size-selective sieving of nanoparticles using a highly oriented two-dimensional supramolecular polymer. *Angew. Chem. Int. Ed.* **59**, 4840−4845 (2020).

18 Guo, X. *et al.* Metal-organic polyhedron membranes for molecular separation. *J. Membr. Sci.* **632**,119354 (2021).

19 Wang, X. *et al.* Robust ultrathin nanoporous mof membrane with intra-crystalline defects for fast water transport. *Nat. Commun.* **13**, 266 (2022).

20 Cheng, P. *et al.* Two-dimensional metal−porphyrin framework membranes for efficient molecular sieving. *J. Membr. Sci.* **640**, 119812 (2021).

21 Zhang, R. *et al.* Coordination-driven in situ self-assembly strategy for the preparation of metal-organic framework hybrid membranes. *Angew. Chem. Int. Ed.* **53**, 9775−9779 (2014).

22 He, A. *et al.* A smart and responsive crystalline porous organic cage membrane with switchable pore apertures for graded molecular sieving. *Nat. Mater.*, https://doi.org/10.1038/s41563-41021-01168-z (2022).

23 Khan, N. A. *et al.* Solid-vapor interface engineered covalent organic framework membranes for molecular separation. *J. Am. Chem. Soc.* **142**, 13450−13458 (2020).

24 Dey, K. *et al.* Selective molecular separation by interfacially crystallized covalent organic framework thin films. *J. Am. Chem. Soc.* **139**, 13083−13091 (2017).

25 Fan, H., Gu, J., Meng, H., Knebel, A. & Caro, J. High-flux membranes based on the covalent organic framework COF-LZU1 for selective dye separation by nanofiltration. *Angew. Chem. Int. Ed.* **57**, 4083−4087 (2018).

26 Zhang, W. *et al.* General synthesis of ultrafine metal oxide/reduced graphene oxide nanocomposites for ultrahigh-flux nanofiltration membrane. *Nat. Commun.* **13**, 471 (2022).

27 Yang, Q. *et al.* Ultrathin graphene-based membrane with precise molecular sieving and ultrafast solvent permeation. *Nat. Mater.* **16**, 1198−1202 (2017).

28 Shen, Y.-x. *et al.* Achieving high permeability and enhanced selectivity for angstrom-scale separations using artificial water channel membranes. *Nat. Commun.* **9**, 2294 (2018).

29 Zhou, Z. *et al.* Electropolymerization of robust conjugated microporous polymer membranes for rapid solvent transport and narrow molecular sieving. *Nat. Commun.* **11**, 5323 (2020).

30 Huang, T., Puspasari, T., Nunes, S. P. & Peinemann, K. V. Ultrathin 2D-layered cyclodextrin membranes for high-performance organic solvent nanofiltration. *Adv. Funct. Mater.* **30** (2019).

31 Tang, M. J. *et al.* Precisely patterned nanostrand surface of cucurbituril[n]-based nanofiltration membranes for effective alcohol-water condensation. *Nano Lett.* **20**, 2717−2723 (2020).

32 Wang, H. *et al.* Membrane adsorbers with ultrahigh metal-organic framework loading for high flux separations. *Nat. Commun.* **10**, 4204 (2019).

33 Chen, T. *et al.* Highly crystalline ionic covalent organic framework membrane for nanofiltration and charge-controlled organic pollutants removal. *Sep. Purif. Technol.* **256**,117787 (2021).

34 Huang, T. *et al.* Molecularly-porous ultrathin membranes for highly selective organic solvent nanofiltration. *Nat. Commun.* **11**, 5882 (2020).

35 Yang, F., Tao, F., Li, C., Gao, L. & Yang, P. Self-assembled membrane composed of amyloid-like proteins for efficient size-selective molecular separation and dialysis. *Nat. Commun.* **9**, 5443 (2018).

36 Qiu, X. *et al.* Selective separation of similarly sized proteins with tunable nanoporous block copolymer membranes. *ACS Nano* **7**, 768−776 (2013).

37 Zhang, Z. *et al.* Hybrid organic-inorganic-organic isoporous membranes with tunable pore sizes and functionalities for molecular separation. *Adv. Mater.* **33**, e2105251 (2021).

38 Ku, J.-R. & Stroeve, P. Protein diffusion in charged nanotubes: “On-Off” behavior of molecular transport. *Langmuir* **20**, 2030−2032 (2004).

39 Li, Q. Y., Cui, Z. F. & Pepper, D. S. Fractionation of HSA and IgG by gas sparged ultrafiltration. *J. Membr. Sci.* **136**, 181−190 (1997).

40 Xu, Y., Sleigh, R., Hourigan, J. & Johnson, R. Separation of bovine immunoglobulin G and glycomacropeptide from dairy whey. *Process Biochem.* **36**, 393−399 (2000).
