# Supplementary material for: Supramolecular framework membrane for precise sieving of small molecules, nanoparticles and proteins
Source: Nat Commun. 2023 Feb 22;14:975. doi: 10.1038/s41467-023-36684-w (PMC9944550; doi:10.1038/s41467-023-36684-w)
Supplement: Supplementary file 2 — Description of Additional Supplementary Files [file 41467_2023_36684_MOESM2_ESM.pdf]

Description of Additional Supplementary Files

File Name: Supplementary Movie 1
Description: Filtration of AuMPA NPs.

File Name: Supplementary Movie 2
Description: Filtration of dye MB.

File Name: Supplementary Movie 3
Description: Filtration of dye EY.

File Name: Supplementary Movie 4
Description: Filtration of dye MB&EY
